# Functional Feeds as Immunostimulants for Aquaculture: A Molecular Perspective

**DOI:** 10.1155/anu/2220437

**Published:** 2026-07-03

**Authors:** Uzeme Precious Aluta, Nada Ćujić Nikolić, Arzu Yildirim, Ivana Giovanna Zupičić, Perveen Akhtar, Vladimir Radosavljevic, Sofia A. Costa Lima

**Affiliations:** ^1^ Department of Fisheries and Aquaculture, Faculty of Life Sciences, University of Lagos, Lagos, Nigeria, unilag.edu.ng; ^2^ Institute for Medicinal Plant Research Dr. Josif Pančić, Tadeuša Košćuška 1, Belgrade, Serbia; ^3^ Department of Bioengineering, Faculty of Engineering, Ege University, İzmir, Türkiye, ege.edu.tr; ^4^ Croatian Veterinary Institute, Savska Cesta 143, Zagreb, Croatia, veinst.hr; ^5^ School of Food Science and Environmental Health, Technological University Dublin, Grangegorman, D07ADY7, Dublin, Ireland; ^6^ Institute of Veterinary Medicine of Serbia, Janisa Janulisa 14, 11000, Belgrade, Serbia; ^7^ LAQV-REQUIMTE, School of Medicine and Biomedical Sciences of the University of Porto, Rua Jorge Viterbo Ferreira 228, Porto, Portugal

**Keywords:** aquaculture, dietary immunostimulants, disease resistance, immune response, molecular approaches

## Abstract

The development of aquafeeds that enhance immunity in fish represents an effective strategy for disease management in aquaculture. This approach involves incorporating functional ingredients with immunostimulatory properties to improve fish welfare. The use of dietary immunostimulants is considered a suitable, safe, and eco‐friendly alternative to mitigate the adverse effects of antibiotics and chemotherapeutics. Hence, there is growing interest among aquaculture stakeholders in immunostimulant sources such as algae, plant derivatives, trace minerals, prebiotics, and probiotics. However, earlier studies have relied on biochemical markers to evaluate the interactions of dietary immunostimulants with the fish immune system, which provide limited mechanistic insights and reduce reproducibility for large‐scale applications. Recent advances in molecular approaches, including genomics, transcriptomics, proteomics, metabolomics, and small RNA profiling, have been increasingly applied to elucidate the underlying mechanisms of dietary immunostimulants and to guide the development of species‐specific formulations. By integrating these molecular tools, researchers can design sustainable, species‐tailored immunostimulant diets that reduce the reliance on antibiotics. This review synthesizes molecular evidence on nutrient‐, plant‐, microbial‐, and algae‐based immunostimulants, highlighting how omics tools elucidate their mechanisms and support the design of species‐specific functional feeds.

## 1. Introduction

**Figure 1:**
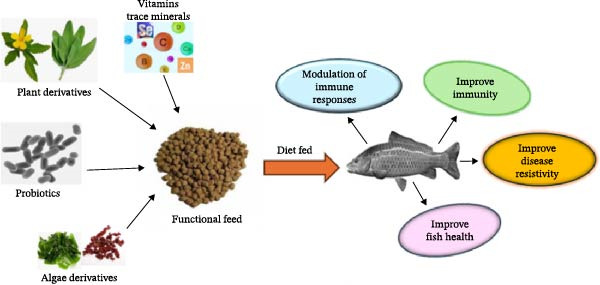
Development and application of functional feeds in fish health management.

Aquaculture supports global food security by providing ~51% of fish for human consumption and nutrition [1]. With continuous intensified fish production, aquaculture is projected to be the major source of aquatic animal proteins for the growing global population [2]. To meet this demand, there is a need to implement innovative strategies that address concerns centered on aquatic animal health and well‐being since diseases are a major limiting factor to the sustainable development of the aquaculture industry [3]. At the forefront of these innovative strategies is the application of functional feeds, designed to go beyond basic nutritional needs, which play a crucial role in promoting health and improving resistance to disease in aquaculture species (Figure 1). Thus, the concept of immunostimulants represents a pragmatic approach to support fish immunity and provide effective protection against pathogen invasion.

Disease outbreaks account for up to 40%–60% of production losses in some aquaculture systems, leading to billions of dollars in annual economic impact worldwide [4, 5]. Conventional strategies such as antibiotics and chemotherapeutics remain important tools in disease management, although concerns regarding antimicrobial resistance and environmental safety have emerged, particularly in cases of inappropriate or excessive use. Vaccination, while effective for certain species and pathogens, is not always feasible in diverse, small‐scale aquaculture settings. This has driven interest in functional feeds that not only meet nutritional requirements but also enhance immune resilience through bioactive compounds.

Functional feeds act as immunostimulants by stimulating the innate and adaptive immune systems, either through the supply of dietary micronutrients such as vitamins, trace minerals, amino acids, fatty acids, etc., or nonnutritive ingredients such as probiotics, prebiotics, phytogenics, and antimicrobial peptides (AMPs) with known immunostimulatory traits [6]. The immunostimulating components in the fish feed are made up of molecules that mimic pathogen‐associated molecular patterns (PAMPs) and bind with pattern recognition receptors (PRRs) such as Toll‐like receptors (TLRs), NOD‐like receptors (NLRs), and C‐type lectins. This interaction precedes the production of signaling molecules such as cytokines, which initiate innate immune responses and promote adaptive changes in the lymphoid system required to eradicate the pathogen and sustain immunity [7, 8]. For instance, β‐glucan, a microbial‐derived PAMP often administered through diet, has been widely studied for its direct stimulation of the immune system and enhancement of disease resistance [9–12].

Despite their promise, most studies to date have evaluated functional feeds using biochemical assays of immune activity, which only provide a partial picture of their effects. To fully realize the potential of immunostimulant feeds, it is essential to integrate molecular approaches that reveal the underlying mechanisms of action. Up until recently, evaluating the role of functional feeds in fish systemic immunity using molecular techniques has not been a routine practice. Most research findings are often based on biochemical analyses of immunological parameters such as lysozyme, respiratory burst, myeloperoxidase, complement, phagocytic activities, immunoglobulin levels, and oxidative stress enzymes, etc. These biochemical markers only provide an indication of the effects of the assessed dietary immunostimulant without elucidating its mechanisms of action and molecular pathways. However, the current advancements in biotechnology have made it easier to assess the effects of functional feed formulations on immune‐related biological molecules (DNA, RNA, proteins, and metabolites) in fish species. These molecular approaches have to some extent given insights into the interactions of dietary immunostimulants and the fish immune system, hence paving the way for the development and application of functional feeds as a safe and sustainable prophylaxis for aquaculture. Therefore, this work aims to comprehensively review the immunostimulatory effects of several functional ingredients integrated into fish feeds from a molecular perspective.

## 2. Literature Research Strategy

**Figure 2:**
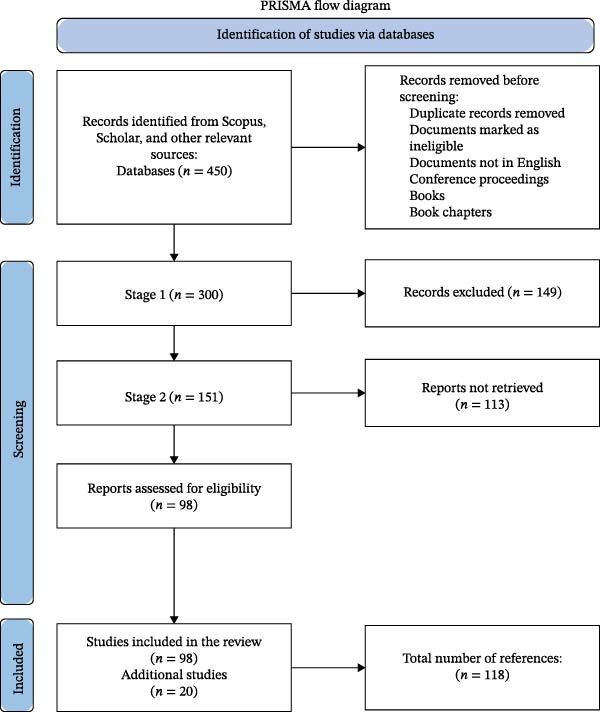
PRISMA diagram with the representation of the literature screening and inclusion of relevant studies.

The development of this manuscript was made through author collaboration within COST Action BioAqua, which supports scientific networking across Europe. The literature search was conducted electronically using Scopus, Scholar, Medline, PubMed, and Web of Science databases in the period of January to [13]. The selected databases for this review offer comprehensive and multidisciplinary coverage of peer‐reviewed journal articles across relevant disciplines. In line with the established guidance on systematic review methodology, these databases provided a robust search functionality, including advanced evidence search options. The research was limited to peer‐reviewed and published journal articles only, written in English, and other literature was not considered for inclusion. The keywords relevant to the review paper were used during the search strategy. Inclusion criteria were used throughout the search, shortlisting, and selection of the relevant evidence. The literature identification and selection process is presented through a structured flow scheme, presented using the PRISMA diagram, that illustrates the screening and inclusion of relevant publications (Figure 2). The review follows a narrative approach, enriched with selected systematic features, including database‐driven literature searches and clearly defined inclusion criteria. Nevertheless, the study does not fully comply with formal PRISMA requirements, such as protocol registration, exhaustive search documentation, formal risk‐of‐bias assessment, or complete adherence to standardized reporting elements. Accordingly, this work should be regarded as a narrative review with a methodological structure rather than a fully systematic review. The included flow scheme is adapted from PRISMA principles to enhance transparency in the literature selection process without implying full methodological conformity.

## 3. Integration of Molecular Tools for Understanding Fish Immune Responses to Functional Feed

**Figure 3:**
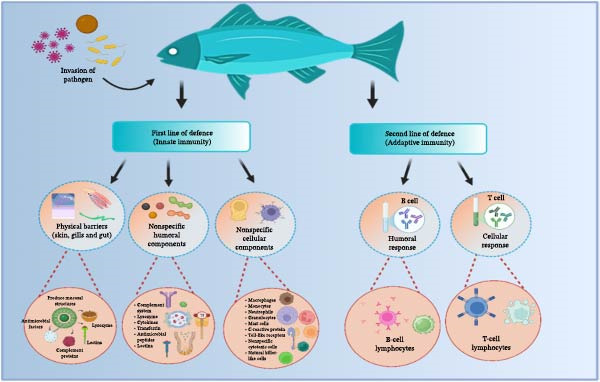
Fish immune system and components (created with BioRender.com).

The innate immune system (nonspecific) and the adaptive immune system (specific) are the two components of the fish immune system (Figure 3). The innate immunity acts as the first line of defense and comprises physical barriers such as gills, gut, and skin that produce mucosal structures that consist of antimicrobial factors and other potent molecules such as lysozyme, lectins, complement proteins, and immunoglobulins. This first line of defense includes nonspecific humoral components (cytokines, complement system, transferrin, and AMPs) and nonspecific cellular components (neutrophils, macrophages, natural killer‐like cells, and monocytes) [14]. Additionally, the complement system comprises 30 inactive circulating proteins and membrane‐bound receptors that can be activated through classical pathways, that is, activated by antigen‐antibody complexes, the alternative pathway, which is activated by molecules on microorganism surfaces, and lectin pathways. However, the adaptive cell‐mediated immune system, the second line of defense, is activated after primary defense, with B lymphocytes producing antibodies (the humoral response) and T lymphocytes mediating cellular responses. While fish possess both systems, innate immunity plays the more dominant role and provides fish with the ability to resist pathogenic agents [15].

**Figure 4:**
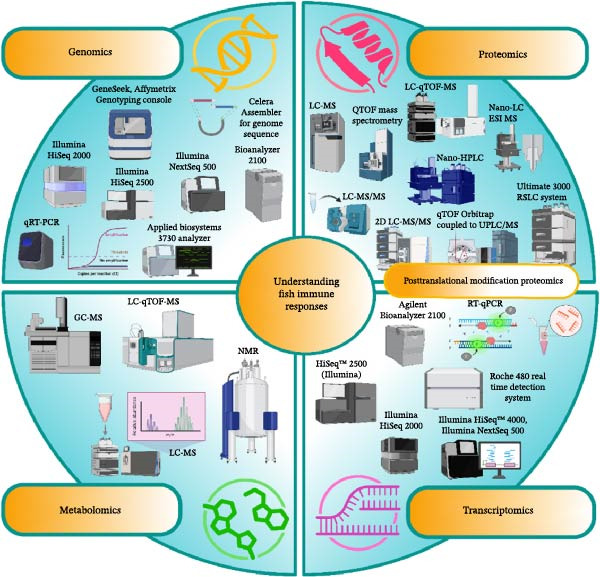
Molecular tools for understanding fish immune responses. Key platforms utilized in genomics, proteomics, posttranslational modifications, metabolomics, and transcriptomics (created with BioRender.com).

Understanding the complex interactions between functional feed components and the fish immune system requires advanced molecular tools (Figure 4) capable of dissecting immunological processes at the cellular and gene levels. Traditional performance‐based endpoints, such as growth and survival, while informative, do not reveal the mechanistic basis of feed‐induced immunomodulation. Over the past two decades, molecular biology techniques such as quantitative real‐time PCR (qRT‐PCR), transcriptomics, proteomics, metabolomics, and cell‐based assays have transformed our understanding of how functional feeds, including β‐glucans, yeast derivatives, and probiotic metabolites, modulate immunity in aquaculture species [16, 17]. These molecular insights are critical for defining immune signatures, developing biomarkers of health, and designing precision functional feeds that reduce antibiotic dependance and improve sustainability [18, 19].

Additionally, advances in omics technologies such as transcriptomics (RNA sequencing [RNA‐seq] and microarray), proteomics, metabolomics, and microbiome profiling are providing new insights into how nutrition and feed impact fish immunity. Through molecular pathway enrichment analysis, the genes involved can reveal how immune functions are influenced by the feed. Studies have shown that gene expression profiling, protein expression, and metabolites with microbiomics approaches provide the functional feed‐induced impact on fish immunity, and immune reactions can be fully understood by high‐throughput approaches [16].

Functional genomics has become a pivotal approach in aquaculture to enable comprehensive insights into the molecular mechanisms underlying immune response, growth, stress tolerance, and reproductive efficiency, and detailed analysis of gene expression and regulation in response to functional feeds [20]. qRT‐PCR is a widely used method for assessing immune‐related gene expression in fish tissues. Genes commonly monitored include proinflammatory cytokines (interleukin [*IL-1*B], tumor necrosis factor‐alpha [*TNF*‐A]), anti‐inflammatory mediators (interleukin‐10 [*IL-10*]), AMPs (*hepcidin*,*β-defensins*), and transcription factors such as nuclear factor kappa B (*NF-KB*) and *JAK/STAT*. Dietary supplementation with β‐glucans has consistently been linked to the upregulation of these immune markers and improved disease resistance in tilapia, rainbow trout, and salmonids [21, 22]. Studies have demonstrated that functional feeds can modulate immune responses in multiple fish species, including gilthead seabream, Atlantic salmon, Nile tilapia, rainbow trout, Siberian sturgeon, and Asian seabass. Moreover, molecular tools such as microarray, in situ hybridization (ISH), RNA‐seq, qPCR, and small RNA‐seq have revealed feed‐dependent differential expression of immune‐related genes (e.g., T and B cell responses, selenoproteins, cytokines, lncRNAs, and miRNAs) in key tissues, including gills, liver, intestine, head, heart, and kidney [2, 23–27]. Moreover, integration of transcriptomic, epigenetic, and genomic data has provided insights into novel pathways of the fish immune response modulated by functional feeds, including PI signaling, LC‐PUFA biosynthesis, glycolysis, interferon pathways, and immune‐inflammatory responses [25].

Transcriptomic tools, particularly RNA‐seq, allow for high‐resolution analysis of gene expression across the whole transcriptome and have advanced the understanding of fish responses to functional feeds. Transcriptomic studies demonstrate that functional feeds modulate immune pathways including cytokine signaling, antigen presentation, oxidative stress responses, and complement activation [16]. In Atlantic salmon, transcriptomic profiling of the liver and head kidney revealed that prebiotic‐containing diets reprogram immune and metabolic gene expression, enhancing disease resistance [17]. In zebrafish, β‐glucan supplementation upregulated *il-8*, *TNF-A*, and AMP genes in gut epithelial cells, capturing localized mucosal immune modulation [21]. Furthermore, early‐life transcriptomic studies show that colonization by beneficial microbiota during larval stages primes innate immune pathways, shaping adaptive immunity later in life [26]. Moreover, transcriptomic studies have advanced the understanding of fish responses to functional feeds and fishmeal replacements as functional feeds, including immunostimulants, prebiotics, probiotics, GABA, plant extracts, and fatty acids (ω3 LC‐PUFA) were shown to influence gene expression linked to inflammation, antibacterial responses, antiviral responses, oxidative stress, intestinal homeostasis, and lipid metabolism [25, 28]. Vallejos Vidal [29] conducted a study using both molecular (transcriptomics) and cellular assays to evaluate gene expression profiles linked with exposure to immunostimulants over time in fish gills. Several transcriptomic and qRT‐PCR studies have shown that dietary selenium, vitamins C and E, and omega‐3 fatty acids modulate the expression of antioxidant enzymes (superoxide dismutase [SOD], catalase [CAT], and glutathione peroxidase [GPX]) and pro‐/anti‐inflammatory cytokines, confirming the mechanisms proposed for nutrient‐based immunostimulants.

Proteomics, using mass spectrometry and gel‐based separations, provides insights into protein‐level changes in response to functional feeds. Dietary β‐glucans and yeast derivatives have been shown to alter complement proteins, lectins, heat shock proteins, and immunoglobulins in fish, consistent with enhanced innate immunity [21, 22]. Additionally, proteomic analyses reveal how dietary interventions affect fish growth, metabolism, and immune responses. For instance, Ghaedi et al. [30] demonstrated that dietary β‐glucan (0.2%) enhanced growth and feed efficiency, altering muscle proteins by upregulating tropomyosins and downregulating myosins, suggesting a link to improved growth in *Oncorhynchus mykiss*.

Metabolomics complements these findings by mapping diet‐induced metabolic shifts associated with immune activation, including alterations in lipid mediators, reactive oxygen species (ROS) intermediates, and antioxidant metabolites [31]. Importantly, yeast cell wall polysaccharides (β‐glucans, mannans, and chitin) have been linked to improved redox homeostasis, stress tolerance, and growth, reinforcing their role as sustainable immunostimulants in aquaculture [22]. Furthermore, metabolomics provides insights into the components of feed and their impact on fish metabolism, linking nutrition, physiology, and growth through nontargeted analyses of metabolites in biofluids and tissues using NMR and MS [32]. High‐throughput techniques like GC–MS and NMR also characterize gut microbiome metabolites to optimize functional feeds [13].

In addition to transcriptomic and proteomic profiling, posttranslational modifications (PTMs) such as phosphorylation, ubiquitination, acetylation, methylation, and glycosylation can also reveal regulatory changes in immune responses to functional feeds. PTM profiling, such as ubiquitin‐enriched proteomics and phosphoproteomics, can add mechanistic depth to functional feed studies where regulatory changes are not apparent from total protein levels or mRNA alone, as different components of functional feed, such as β‐glucans, can influence immune signaling not only by changing expression levels but also by affecting the phosphorylation state or other PTMs of key signaling proteins [33].

These molecular tools have been successfully applied to characterize nutrient‐, plant‐, microbial‐, and algae‐based immunostimulants, enabling pathway‐level mapping of their effects on fish immunity.

## 4. The Functionality of Dietary Immunostimulants in Fish Species From a Molecular Perspective

Dietary immunostimulants, used as feed additives, play a crucial role in supporting fish health and promoting growth by enhancing resistance to infectious diseases in aquaculture [34, 35]. These compounds, whether natural or synthetic, are commonly incorporated into fish diets to stimulate immune responses (Table 1). They primarily stimulate components of the innate immune system, including macrophages, neutrophils, and the production of immune‐related molecules such as gamma‐interferon [36].

**Table 1:** Main categories of dietary immunostimulants, examples, and mode of action.

Category	Examples	Mode of action
Polysaccharides	β‐glucans, chitosan, and mannans	Bind to immune cell receptors (TLRs and dectin‐1), activate cytokine production
Nucleotides	Purine/pyrimidine‐rich yeast extracts	Support immune cell proliferation and antibody synthesis
Herbal extracts	Garlic (allicin) and turmeric (curcumin)	Antioxidant, anti‐inflammatory, and immunomodulatory properties
Probiotics	*Lactobacillus*, *Bacillus*, and *Saccharomyces*	Improve gut immunity, stimulate the mucosal immune system
Synthetic compounds	Levamisole and CpG oligonucleotides	Direct activation of immune receptors promotes T and B cell activity

In fish, the innate immune system serves as the first and most rapid line of defense, capable of responding to pathogens within hours. This quick response is especially critical in aquaculture settings, where high stocking densities and frequent pathogen exposure increase the risk of disease outbreaks [37]. The resilience of the innate immune system under stress, combined with its ability to be enhanced through dietary immunostimulants, a concept referred to as immune training, and proper environmental management, is essential for maintaining health and preventing disease in intensive aquaculture systems [38]. Dietary immunostimulants enhance fish immune responses not only at the cellular level but also through specific molecular mechanisms. These bioactive compounds modulate innate and adaptive immunity by interacting with immune receptors, altering gene expression, and activating key signaling pathways involved in host defense, as briefly presented in Table 2. By enhancing immune responsiveness, these feed additives help fish better withstand pathogen exposure and environmental stressors typical of intensive aquaculture systems. This strategy not only reduces dependance on antibiotics but also supports improved growth performance and fosters more sustainable and resilient aquaculture practices.

**Table 2:** Modulation of innate and adaptive immunity by dietary immunostimulants.

Type of mechanism	Targets
Activation of pattern recognition receptors (PRRs)	Toll‐like receptors (TLRs), NOD‐like receptors (NLRs), and C‐type lectins are key PRRs found in fish immune cells. For example, β‐glucans can bind to TLR2 and TLR4 on the surface of immune cells, thereby triggering downstream signaling cascades that initiate defensive responses.
Immune‐related gene expression	Proinflammatory cytokines: genes such as interleukin‐1β (IL‐1B), tumor necrosis factor‐alpha (TNF‐A), and interferon‐gamma (IFN‐γ), are commonly upregulated, promoting an inflammatory response to help eliminate pathogens. Antimicrobial peptides (AMPs): the expression of genes coding for AMPs, such as hepcidin and defensins, is often enhanced, contributing directly to microbial killing.
Activation of signal transduction pathways	NF‐KB pathway: controls the transcription of numerous immune genes involved in inflammation and pathogen defense. MAPK (mitogen‐activated protein kinase) pathway: regulates cell differentiation, inflammation, and survival. JAK/STAT (Janus kinase/signal transducers and activators of transcription) pathway: transduces cytokine signals to promote the expression of immune effector molecules.
Activation and modulation of immune cells	Macrophages and neutrophils often show increased phagocytic activity and elevated production of reactive oxygen species (ROS), which are essential for destroying engulfed pathogens. Antigen presentation is enhanced via upregulation of major histocompatibility complex (MHC) molecules on antigen‐presenting cells, facilitating better activation of lymphocytes.

### 4.1. Nutrient‐Based Immunostimulants: Molecular Mechanisms and Biomarkers

Nutrient‐based dietary immunostimulants are key feed components in aquaculture that go beyond meeting basic nutritional requirements and actively support and regulate the immune system of cultured species. These include specific vitamins, minerals, amino acids, and fatty acids that enhance immune responses at both the cellular and molecular levels. Incorporating these nutrients into aquafeeds helps prevent disease, promote growth, and reduce reliance on chemotherapeutic treatments, which is especially important in intensive farming systems. Unlike pharmacological agents, these nutrients function by supporting essential physiological processes while simultaneously activating key immune pathways, particularly those involved in the innate immune response. Their immunomodulatory actions work through various mechanisms, such as strengthening antioxidant defenses, regulating cytokine production, stimulating immune cell activity, and reinforcing mucosal barriers. Additionally, they influence critical molecular signaling pathways, including NF‐KB, mitogen‐activated protein kinase (MAPK), and JAK/STAT (Table 2). As a result, nutrient‐based immunostimulants enhance the overall health and resilience of aquaculture species, contributing to more sustainable and productive farming practices [39].

Minerals are indispensable in numerous physiological processes across animal species, including fish [40]. Of the ~90 natural elements, 22 are considered essential for life [41]. Fish possess unique physiological mechanisms that allow them to absorb and retain minerals both from their diet and directly from the aquatic environment [40]. Mineral supplementation in aquafeeds has been shown to promote growth, enhance nonspecific immunity, and improve disease resistance [42]. Trace minerals such as selenium (Se) and zinc (Zn) and vitamins such as C and E are key cofactors for major antioxidant enzymes, including SOD, CAT, and GPX [43]. These enzymes protect immune cells from damage caused by ROS, which increase during inflammation and pathogen exposure. The combined use of Se, vitamin C, and vitamin E strengthens antioxidant defenses and supports immune health in aquaculture species, enhancing both growth and disease resistance [44].

Vitamins A and E also help maintain the integrity of epithelial cells, thereby supporting mucosal barriers in the skin, gills, and gastrointestinal tract, which serve as key frontline defenses that prevent pathogen invasion and trigger local immune responses on contact. Micronutrients such as zinc and vitamin C are shown to enhance the phagocytic activity of immune cells like macrophages and neutrophils, improving the recognition and elimination of pathogens. The amino acid arginine further boosts immune cell microbicidal capacity by serving as a precursor for nitric oxide (NO) synthesis, which aids in pathogen destruction.

By maintaining the redox balance, these nutrients enhance immune cell survival and efficiency. Specific amino acids (e.g., arginine and glutamine) and fatty acids (e.g., EPA/DHA) regulate the expression of cytokines such as IL‐1B, TNF‐A, and IFN‐γ, which orchestrate inflammation, recruit immune cells, and facilitate pathogen clearance. Omega‐3 fatty acids, in particular, can suppress excessive inflammation by downregulating proinflammatory genes via the NF‐KB pathway.

Nucleotides, which are low‐molecular‐weight intracellular compounds, are crucial for nearly all biochemical processes in both vertebrates and invertebrates [45]. Although they are not essential nutrients under normal circumstances due to endogenous synthesis, dietary nucleotides become important under stress, nutrient deficiency, injury, or early developmental stages [46]. Consequently, they are often regarded as conditionally essential [47]. For instance, Bae et al. [48] demonstrated that dietary inosine monophosphate (IMP), combined with shrimp soluble extract (SSE), enhanced growth, immunity, and disease resistance in juvenile Nile tilapia. Importantly, both IMP and SSE can be derived from waste valorization processes, making their use economically viable and sustainable. Similarly, Choi et al. [49] found that dietary nucleotides extracted from yeast, when combined with probiotics, could serve as an alternative to antibiotics in juvenile olive flounder by improving growth performance, immune response, gut health, and disease resistance.

Finally, nucleotides, vitamin A, and glutamine are crucial for the proliferation and differentiation of T and B lymphocytes, promoting robust adaptive immune responses and supporting mucosal immunity within the gut‐associated lymphoid tissue (GALT), an essential defense for aquatic species constantly exposed to waterborne pathogens. These mechanisms have been increasingly validated through genomics, transcriptomics, proteomics, and metabolomics, which reveal how specific nutrient supplements reshape immune gene networks and metabolic pathways (Table 3). These molecular approaches are detailed in Section 3.

**Table 3:** Nutrient‐based immunostimulants: molecular targets and validated biomarkers.

Nutrient‐based immunostimulants	Key molecular targets/pathways (see Table 2)	Validated molecular biomarkers/tools (Section 3)
Vitamins C, E; trace minerals (Se, Zn)	Antioxidant and redox regulation; NF‐KB, MAPK, and Nrf2 signaling	Enzyme activity: SOD, CAT, GPX; qRT‐PCR: *SOD*, *CAT*, *GPX*; ROS/MDA; transcriptomics
Vitamin A	Epithelial integrity; mucosal immunity; lymphocyte differentiation; cytokine balance	qRT‐PCR: *IL-10*, *TGF-B*; mucosal genes; gut histology; transcriptomics
Arginine and glutamine	Immune cell metabolism; NO synthesis; JAK/STAT signaling; T/B cell proliferation	qRT‐PCR: *inos*, *IL-1B*, *TNF-A*, *CD4*, *cd8*, IGM; NO assays; cell‐based assays
Omega‐3 fatty acids (EPA/DHA)	Inflammatory modulation; eicosanoid synthesis; NF‐KB suppression	qRT‐PCR: *IL-1B*, *TNF-A*, *ifn-γ*; lipid mediator profiling; transcriptomics, metabolomics
Dietary nucleotides	Immune cell proliferation; innate–adaptive immune crosstalk; gut immunity	qRT‐PCR immune panels; RNA‐seq; growth and challenge biomarkers; microbiome profiling
Combined micronutrients	Integrated regulation of NF‐KB, MAPK, and JAK/STAT pathways	Multiomics integration (RNA‐seq, proteomics, and metabolomics); pathway enrichment

*Note:* Molecular targets and biomarkers are interpreted in the context of fish innate and adaptive immune components (Figure 3) and the molecular and omics‐based analytical platforms used to assess functional feed–immune interactions (Figure 4).

### 4.2. Plant‐Based Immunostimulants: Molecular Mechanisms and Biomarkers

The significance of plants and their derivatives as immunostimulants has received global attention, placing it at the forefront of active investigations in aquaculture research. Various plants and their derivatives have been incorporated into animal feed, demonstrating notable health‐promoting effects in farmed fish while remaining safe for the environment and public health. Several plant bioactive compounds have been shown to modulate fish immune responses and improve disease resistance against infectious microbes. However, their efficacy has largely been evaluated by serological analysis of nonspecific cellular and humoral innate immune parameters and cumulative mortality against pathogens. Over the past decade, efforts have been intensified to evaluate the modulations of molecular biomarkers associated with fish immune responses to plant‐based immunostimulant diets (Table 4). Plant derivatives such as polyphenols, terpenoids, essential oils, pigments, polysaccharides, and AMPs have been reported for their effectiveness in triggering a cascade of signaling pathways that leads to the activation of transcription factors involved in the regulation of immune genes [68–70].

**Table 4:** Molecular findings of selected plant‐based immunostimulants in fish species.

Plant‐based immunostimulants	Fish species	Inclusion level	Feeding period	Tissue analyzed	Molecular findings	References
*Vitex agnus-castus* extract	Goldfish (*Carassius auratus)*	5, 10, and 15 g/kg	56 days	Anterior kidney	Upregulated mRNA levels of TNF‐A and TNF‐2α	[50]
*Salvia officinalis* and *Lippia citriodora* leaf extract	Gilthead seabream (*Sparus aurata)*	0.5%	92 days	Spleen	Upregulated TNF‐A, IL‐1B, IL‐10, lysozyme, and *CD4*	[51]
*Turnera diffusa* leaf extract	Almaco jack (*Seriola rivoliana)*	0.5%	14 and 28 days	Intestine	Upregulated TNF‐A, IL‐1B, IL‐10, MARCO, and piscidin	[52]
Dandelion root extract	Golden pompano (*Trachinotus ovatus)*	0.5 and 1 g/kg	56 days	Spleen	Upregulated IL‐10 and GPX; nonsignificant effect on TGF‐β1, GRx, and CAT	[53]
Laurel‐leaf cistus ethanolic extract	Common carp (*Cyprinus carpio)*	0.1, 0.5, and 1 g/kg	45 days	Head kidney	Upregulated IL‐1B, IL‐10, IL‐8, and IL‐6	[54]
*Valeriana officinalis* and *Passiflora incarnata* extract (PASSIF MOOD)	Rainbow trout *(Oncorhynchus mykiss)*	470, 940, 1410, 1880, and 2350 mg/kg	21 days	Head kidney	Upregulated lysozyme gene II and IGM. Downregulated TNF‐A and HSP70 genes. Enhanced disease resistance against *Lactococcus garvieae*	[55]
Quercetin	Common carp (*Cyprinus carpio)*	200, 400, and 600 mg/kg	60 days	Liver	Upregulated lysozyme and GPX	[56]
*Ginkgo biloba* leaf extract	*Oreochromis niloticus* (Nile tilapia)	5.0, 7.0, and 9.0 g/kg	8 weeks	Spleen	Upregulated mRNA expression of *IL-1B*, *IL-6*, *IL-10*, *TNF-A*, and *IFN-γ* genes	[57]
*Polygonum minus* extract	Rainbow trout *(Oncorhynchus mykiss)*	5, 10, and 15 mg/kg	8 weeks	Head kidney	Upregulated *IL-1B*, *IL-8*, *TNF-A*, and *lysozyme* genes. Enhanced disease resistance against *Yersinia ruckeri*	[58]
Olive leaf (*Olea europea* L.) extract	Rainbow trout *(Oncorhynchus mykiss)*	0.1, 0.25, 0.5, and 1.0%	60 days	Spleen	Supplementation at 0.1% upregulated *IL-1B*, *IL-8*, *TNF-A*, and enhanced disease resistance against *Yersinia ruckeri*	[59]
*Silybum marianum* extract	*Oreochromis niloticus* (Nile tilapia)	1, 2, 3, and 4 g/kg	60 days	Spleen	Upregulated *IFN-γ1*, *lysozyme*, and hepcidin genes	[60]
*Adansonia digitata* (Baobab) fruit extract	*Oreochromis niloticus* (Nile tilapia)	1, 2, 3, and 4 g/kg	2 months	Liver	Upregulated *IFN-γ1*, *lysozyme*, *IL-1B*, *IL-10*, and hepcidin genes. A dosage of 4 g/kg enhanced disease resistance against *Aeromonas hydrophila*	[61]
*Aloe barbadensis*	Rainbow trout (*Oncorhynchus mykiss)*	5, 10, and 15 g/kg	8 weeks	Kidney	Upregulated TNF‐A, IL‐1B, IL‐6, and IL‐8 gene expression. 15 g/kg led to improved disease resistance against *Saprolegnia parasitica*	[62]
*Berberis vulgaris* extract	Siberian sturgeon (*Acipenser baerii)*	150, 300, 600, and 750 mg/kg	8 weeks	Liver	Downregulated HSP70 and cytochrome p450.	[63]
*Valeriana officinalis* extract	Common carp (*Cyprinus carpio)*	0.25, 0.5, and 1.0%	30 days	Head kidney	Upregulated IL‐1B. Downregulated IL‐10. Nonsignificant effect on TNF‐A	[64]
*Allium sativum*	*Oreochromis niloticus* (Nile tilapia)	1.0 g/kg	14 days	Intestine	Modulates gut microbiota. Upregulated IL‐10 and IL‐17 F. Enhanced resistance against *Streptococcus iniae* infection.	[65]
*Syzygium cumini* leaf extract	Common carp (*Cyprinus carpio)*	10 g/kg	8 weeks	Lymphoid organs	Activated and upregulated NF‐KB, upregulated TNF‐A, and IL‐1B. Improved resistance against *A. hydrophila* infection.	[66]
*Olea europaea* extract (AQUOLIVE)	Atlantic salmon (*Salmo salar)*	0.15%	133 days	Head kidney	Activates I‐kappa B kinase/NF‐kappa signaling. Improved disease resistance against *Aeromonas salmonicida*	[67]

Molecular studies have reported the modulation of inflammatory cytokine genes, which are key regulators of cell‐mediated immunity, in fish fed plant‐based immunostimulant diets. Aquaculture finfish species such as the longfin yellowtail (*Seriola rivoliana*) fed a diet supplemented with *Turnera diffusa* leaf extract at a 0.5% inclusion level showed an upregulation of IL‐1B, IL‐10, TNF‐A, and MARCO (a macrophage receptor gene) [52]. Consequently, there was increased disease resistance against *Aeromonas hydrophila* infections after 28 days of the feeding trial. Rashmeei et al. [50] also found that chasteberry extract caused an increase in mRNA levels of TNF‐A and TNF‐2α in goldfish (*Carassius auratus*) at inclusion levels of 10–15 g/kg of basal diet. The highest expression levels of TNF‐A and IL‐1B were also observed in *Oncorhynchus mykiss* fed diets containing *Cyanus depressus* extract at levels of 1–2 g/kg [71]. Cellular and humoral immune responses were both induced in Atlantic salmon (*Salmo salar*) fed a plant‐based functional diet and subjected to crowding stress [72]. This led to an increase in the gene expression levels of IL‐10, IFN‐γ, CD4, GATA‐3, and IGM, indicating that the functional diet played a protective role against crowding stress in experimental fish. Salomón et al. [51] also reported that gilthead seabream *Sparus aurata* fed a diet supplemented with 0.1% medicinal plant leaf extract rich in triterpenes, polyphenols, and glycosides showed upregulation of genes (TNF‐A, IL‐1B, IL‐10, lysozyme, and IGM) that mediate cellular and humoral immunity. Furthermore, Darvishi et al. [73] demonstrated that an increase in the gene expression levels of cellular and humoral factors may have contributed to the enhanced disease resistance observed in *Oncorhynchus mykiss* against *Yersinia ruckeri* infection after administration of a plant‐based functional diet. Some plant extracts have also been found to downregulate the expression of specific immune‐related genes, which can confer benefits in regulating inflammatory responses. A recent study has shown that proinflammatory cytokines like TNF‐A were downregulated in Nile tilapia (*Oreochromis niloticus*) fed diets containing an aqueous extract of *Laurus nobilis* [74]. In addition, Yilmaz et al. [64] reported that the *Valeriana officinalis* extract had a nonsignificant effect on TNF‐A and significantly reduced IL‐10 expression in *Cyprinus carpio*. The disparities in study outcomes may arise from variations in plant species, extract preparation, phytochemical composition, dietary doses, length of administration, trial procedures, and bioassays [75]. However, clarifying the pharmacokinetics of the tested phytochemical may offer a systemic approach to addressing these discrepancies.

Overall, studies reporting the effect of the administration of plant‐based immunostimulants on fish immune responses associate their immune‐modulating and health‐promoting properties with the secondary metabolites present in the plant extracts. However, the specific mechanisms and pathways followed by these plant‐derived bioactive compounds are still poorly described, hence limiting their standardization and application as functional additives in fish feeds. It is commonly postulated that plant extracts could modulate innate and adaptive immune responses by activating PRRs such as the TLRs, subsequently, triggering signaling cascades such as MyD88, activating transcription factors (NF‐KB) and MAPKs, and initiating inflammatory responses (upregulation/downregulation of cytokines) [76–78]. There is now increasing evidence suggesting that the inhibition of TLR‐mediated NF‐KB signaling pathways could be responsible for the anti‐inflammatory properties of plant‐derived bioactive compounds. For instance, transcriptomic analysis of the hepatic tissue of *Oreochromis niloticus* fed *Radix bupleuri* extract (RBE) (1 and 3 g/kg diet) shows that the RBE‐based diet caused significant downregulation of proinflammatory cytokines by inhibiting the TLRs‐MyD88‐NF‐KB signaling pathway [79]. In *Salmo salar* fed a plant‐based immunostimulant diet (prepared with polyphenols and triterpenic‐rich extract from *Oleo europaea*), transcriptomic profiling of the head kidney revealed the stimulation of i‐kB kinase/NF‐KB signaling pathways and expression of several genes associated with innate and adaptive immunity [67].

### 4.3. Microbial‐Based Immunostimulants: Molecular Mechanisms and Biomarkers

Microbial‐based immunostimulants have emerged as a promising category of functional feed additives in aquaculture, offering safe and sustainable alternatives to chemotherapeutics and antibiotics [80]. This group includes functional molecules derived from bacteria, fungi (including yeasts), and viruses [81]. These compounds comprise β‐glucans (originating from yeast, fungi, and bacteria), lipopolysaccharides (LPS) from Gram‐negative bacteria, muramyl dipeptides (MDP), bacterial DNA, flagellin, and peptidoglycan (PGN) [82], as well as mannan‐oligosaccharides (MOS) and nucleic acids from fungi, and double‐stranded RNA (dsRNA) with its synthetic analog polyinosinic:polycytidylic acid (poly [I:C]) from viruses [83]. Acting through diverse molecular mechanisms, they modulate host immunity and strengthen innate defenses (Table 5). A growing body of experimental work has validated their immunostimulatory potential. In Atlantic salmon, probiotic supplementation with *Carnobacterium divergens* enhanced the gut barrier function and increased survival rates upon challenge with *Vibrio anguillarum* [92]. In zebrafish, yeast β‐glucans were shown to modulate transcriptomic responses in gut epithelial cells, including the upregulation of IL‐8, TNF‐A, and AMP genes [93]. Furthermore, feeding trials with postbiotic metabolites derived from *Lactobacillus plantarum* demonstrated elevated IGM titers and enhanced resistance to *Streptococcus agalactiae* in Nile tilapia (*Oreochromis niloticus*) [94]. Collectively, these findings underscore the value of microbial immunostimulants as functional feed ingredients capable of fine‐tuning host immune responses at both molecular and systemic levels. Upon ingestion, they interact with host PRRs, including TLRs, C‐type lectins, and NLRs, thereby triggering both innate and adaptive immune responses [19, 95]. Through these molecular interactions, microbial immunostimulants activate signaling pathways that regulate cytokine production, complement activity, and AMP synthesis, thereby strengthening the fish immune system against pathogenic challenges [96, 97]. These immunostimulants are commonly incorporated into aquafeeds with the goal of enhancing fish health and resilience [18, 98].

**Table 5:** Microbial‐based immunostimulants in aquaculture: sources, mechanisms, and effects on fish immunity.

Immunostimulant type	Source	Mechanism of action	Effects on fish immunity	Example species	Typical inclusion level and feeding duration
Probiotics	*Lactobacillus*, *Bacillus*, *Enterococcus*, *Shewanella* spp.	Modulate gut microbiota, competitive exclusion, enhance gut barrier, and systemic immunity	Improved phagocytosis, lysozyme activity, respiratory burst, complement activity, and increased WBCs	Tilapia, carp, sea bass, Atlantic salmon, rainbow trout	~ 10^7^–10^9^ CFU/g feed, fed daily for 4–8 weeks; immune effects often measured after ≥4 weeks [84]
Peptidoglycan (PGN)	Bacterial cell walls	Recognized by PGRPs, modulates PAMP accessibility, activates NF‐KB, MAPK, JAK/STAT pathways	Increased phagocytic index, survival rates, and resistance to pathogens	Japanese flounder, orange‐spotted grouper	~ 2.5–10 g/kg feed (0.25%–1.0%) for 4–8 weeks enhances innate immunity [85]
Lipoteichoic acid (LTA)	Gram‐positive bacterial cell walls	TLR2/MyD88 signaling modulates gut microbiota and regulates immune genes	Stimulates antimicrobial peptides, regulates inflammation, and improves resistance to *Streptococcus* spp.	Orange‐spotted grouper	Inclusion often as part of probiotic cell wall fractions; typical trials 30–60 days (exact % often combined) [86]
Lipopolysaccharides (LPS)	Gram‐negative bacterial outer membrane	Recognized by TLRs, activates cytokine production, complement, and antimicrobial peptide synthesis	Enhances phagocytosis, Ig levels, mucus lysozyme activity, and disease resistance	Rainbow trout, Nile tilapia, common carp	Often delivered in challenge studies or injections; dietary inclusion data variable and not standardized [87]
Levan	Microorganisms (*Bacillus* spp., *Zymomonas mobilis*, *Acetobacter xylinum*)	Modulates immune‐responsive and stress‐related genes	Enhances hemoglobin, erythrocyte counts, respiratory burst, phagocytosis, resistance to infections, and stress tolerance	Rohu, common carp, orange‐spotted grouper	Often tested at ~1% diet for 4–8 weeks; specific doses vary widely[88]
Antimicrobial peptides (AMPs)	Bacteria (nisin, subtilin, gramicidin)	Direct bactericidal effect, stimulates host immunity	Reduces pathogen colonization, increases lysozyme and complement activity	Sea bream	Doses depend on peptide; often reported as mg peptide/kg feed in challenge studies (e.g., short periods 2–4 weeks) [89]
β‐glucans	Yeast (*S. cerevisiae*), fungi, bacteria	Recognized by dectin‐1, TLR2; activates Syk, MAPK, NF‐KB	Enhances phagocytosis, cytokine secretion, B lymphocyte differentiation, IGM, IgT/IgZ production	Carp, seabass, tilapia	Typical 0.1%–0.8% diet (1–8 g/kg) for 4–8 weeks; in some studies ≥70 days used for longer effects [90]
Mannan‐oligosaccharides (MOS)	Yeast cell walls	Pathogen‐binding decoy modulates GALT	Improves mucosal immunity, gut health, and growth performance	Various aquaculture species	200–400 mg/kg feed (0.02%–0.04%) for 6–8 weeks stimulates immune genes and lysozyme; combinations with β‐glucan ~1.5–3.0 g kg for 30–60 days also effective [91]

#### 4.3.1. Bacteria‐Derived Immunostimulants

Bacteria‐derived immunostimulants include both probiotics (live beneficial microorganisms) and structural components of bacterial cell walls, such as PGNs, LPS, lipoteichoic acids (LTA), exopolysaccharides, and flagellin. Collectively referred to as PAMPs, these molecules are recognized by host PRRs such as TLRs and NLRs. Their interaction activates downstream signaling cascades (NF‐KB, MAPK, and JAK/STAT), leading to cytokine production, complement activation, phagocytosis, and AMP release. These processes enhance both innate and adaptive immunity, improving resistance to infection [99].

Probiotics such as *Lactobacillus*, *Bacillus*, *Enterococcus*, and *Shewanella* spp. are widely incorporated into aquafeeds to modulate the host microbiota and stimulate immune responses. They exert their effects through multiple mechanisms: competitive exclusion of pathogens, enhancement of gut barrier function, and modulation of systemic immunity. For example, *Bacillus amyloliquefaciens* supplementation improved phagocytic activity, lysozyme production, and survival rates in tilapia (*Oreochromis niloticus*) challenged with *Yersinia ruckeri* [100]. Similarly, *Lactobacillus plantarum* enhanced intestinal immunity and increased resistance to *Vibrio* infections in carp and sea bass [101]. More broadly, probiotic administration has been shown to improve resistance in Atlantic salmon (*Salmo salar*), rainbow trout (*Oncorhynchus mykiss*), and tilapia (*Oreochromis niloticus*) against pathogens such as *A. hydrophila*, *A. salmonicida*, *Edwardsiella tarda*, *Flavobacterium psychrophilum*, *Photobacterium damselae*, and several *Vibrio* spp. [102, 103]. At the physiological level, probiotics enhance innate immune parameters such as lysozyme activity, phagocytosis, respiratory burst, superoxide anion production, serum complement activity, and abundance of macrophages, lymphocytes, erythrocytes, and granulocytes, all of which contribute to robust nonspecific immunity [104]. Among probiotic bacteria, *Bacillus* species, particularly *Bacillus subtilis* and *Bacillus amyloliquefaciens*, are especially valuable due to their spore‐forming ability, heat tolerance, and long shelf life. In tilapia and carp, dietary supplementation with *B. subtilis* upregulated immune‐related genes such as IL‐1B, TNF‐A, and complement while enhancing lysozyme activity and respiratory burst, ultimately improving resistance against bacterial and viral pathogens [92, 105]. Likewise, in zebrafish, *B. amyloliquefaciens* supplementation modulated the gut microbiota composition and promoted antiviral defense through the upregulation of interferon‐stimulated genes [106]. Many of these effects are linked to structural components such as β‐glucans within probiotic cell walls, which are recognized by phagocytic cell receptors. This interaction triggers the release of signaling molecules that stimulate leukopoiesis and cytokine secretion, ultimately increasing white blood cell (WBC) production and strengthening immune readiness [107, 108].

Among bacterial cell wall‐derived immunostimulants, PGN is one of the most studied. As a conserved PAMP, PGN is recognized by PGN recognition proteins (PGRPs), which are evolutionarily conserved molecules with enzymatic functions [32, 109]. Beyond direct recognition, PGN also modulates the accessibility of other PAMPs, thereby amplifying immune signaling. LTA, the main cell wall component of Gram‐positive bacteria, is another important bacterial immunostimulant. LTA interacts with the TLR2 receptor, triggering signaling pathways that regulate immune gene expression and ligand‐binding responses [110]. Dietary supplementation with LTA extracted from *Bacillus pumilus* SE5 activated the TLR/MyD88 signaling pathway in orange‐spotted grouper (*Epinephelus coioides*), modulated the gut microbiota, and stimulated the expression of AMP genes such as epinecidin‐1, hepcidin‐1, and β‐defensin, along with immune‐related genes including TLR2, NOD2, IL‐8, and IGM [111, 112]. Moreover, *Lactobacillus plantarum* LTA has been shown to enhance TLR2‐mediated signaling, while other bacterial components, such as flagellin and LPS, act via TLR5 and TLR9, respectively, reflecting coordinated receptor‐driven immune modulation [113]. Probiotic‐derived LTAs have also been reported to regulate inflammatory mediators, reduce excessive apoptosis, and improve host cell immune responses [80]. Importantly, LTA supplementation has been suggested as a strategy for controlling Gram‐positive bacterial diseases in fish, including *Streptococcus iniae* and *Streptococcus agalactiae* [110].

LPS are major components of the outer membrane of Gram‐negative bacteria, and are considered highly effective immunostimulants even at low doses, with strong immunomodulatory properties in fish [114]. Their administration enhances disease resistance in rainbow trout (*Oncorhynchus mykiss*) and common carp (*Cyprinus carpio*), improving phagocytic activity, elevating serum immunoglobulin levels, and increasing mucus lysozyme activity [115, 116]. In trout, LPS supplementation conferred protection against *A. hydrophila* infection, while in Nile tilapia (*Oreochromis niloticus*), dietary addition of LPS derived from *A. hydrophila* improved resistance to *A. hydrophila* and enhanced survival under stressful environmental conditions [114, 117].

Levan, a fructan‐type exopolysaccharide, is another important bacterial immunostimulant. It is produced by microorganisms such as *Bacillus subtilis*, *Bacillus polymyxa*, *Zymomonas mobilis*, and *Acetobacter xylinum*, as well as some plant species [118]. Dietary supplementation with microbial levan has demonstrated strong immunomodulatory properties in several aquaculture species. In rohu (*Labeo rohita*) infected with *A. hydrophila*, levan supplementation downregulated stress‐related genes (TGF‐B, HSP70) while upregulating immune‐responsive genes (IFN‐γ, β‐2 M, and TLR22), alongside reductions in blood glucose and cortisol [6]. In common carp (*Cyprinus carpio*), microbial levan enhanced hemoglobin levels, erythrocyte counts, respiratory burst activity, and phagocytosis, resulting in greater resistance against infections [119]. Similar benefits were observed in orange‐spotted grouper (*Epinephelus coioides*), where dietary levan improved nonspecific immunity and survival following pathogenic challenge [119]. Additionally, in rohu, levan supplementation enhanced immune resilience and thermal stress tolerance, suggesting broader protective effects under environmental stressors [120].

In addition to structural components and exopolysaccharides, microbial‐derived AMPs have gained attention for their dual antimicrobial and immunostimulatory roles. Well‐known bacterial AMPs such as nisin (*Lactococcus lactis*), subtilin (*Bacillus subtilis*), and gramicidin (*Bacillus brevis*) exert direct bactericidal effects by disrupting pathogen membranes and inhibiting biofilm formation. At the same time, they can activate host immunity. In seabream, dietary supplementation with nisin not only reduced *Vibrio harveyi* colonization but also upregulated lysozyme and complement activity, demonstrating their combined antimicrobial and immunomodulatory potential [121].

#### 4.3.2. Yeast‐Derived Immunostimulants

Yeast and their derivatives are among the most widely applied functional feed additives in aquaculture, with *Saccharomyces cerevisiae* and its byproducts being the most common [122, 123]. Their supplementation has been shown to enhance fish immunity, improve water quality, and increase overall productivity while also reducing the risk of disease outbreaks [124]. Yeast extracts promote the growth of beneficial bacteria and suppress pathogens in the gastrointestinal tract [125]. These effects are largely attributed to yeast cell wall fractions, which contain β‐glucans, mannoproteins, and chitin that provide both nutritional and immunological benefits [126].

Among these components, β‐glucans are the most extensively studied. Acting as PAMPs, they are recognized by dectin‐1 and TLR‐2 on macrophages, neutrophils, and dendritic cells. This recognition initiates phagocytosis, respiratory burst activity, and cytokine secretion, thereby enhancing antigen presentation and stimulating adaptive immune responses [127, 128]. Experimental studies have confirmed these effects in carp, where β‐glucan‐supplemented diets increased lysozyme activity, complement C3 levels, and resistance against *Aeromonas hydrophila* infection [129]. Similar improvements in disease resistance have been reported in seabass and tilapia, with supplementation enhancing survival against *Vibrio anguillarum* and promoting mucosal IgT/IgZ production [130].

Molecular studies unraveled the mechanisms underlying these responses [131]. Following ingestion, β‐glucans are taken up by intestinal enterocytes, potentially mediated by apolipoprotein A‐IV (apoa4), actin (actb), and transgelin (tagln). Recognition by TLR2/6, lectin‐like receptors, and complement receptor 3 (CR3) triggers signaling cascades via spleen tyrosine kinase (Syk), MAPKs, and NF‐KB. This activation leads to the production of cytokines such as IL‐6, IL‐10, and IL‐11, which bridge innate and adaptive immunity, ultimately enhancing B lymphocyte differentiation and immunoglobulin secretion, including IGM and IgT/IgZ, that are critical for mucosal defense in teleosts [96, 97].

Another key yeast‐derived component, MOS, functions as a pathogen‐binding decoy within the gut, preventing colonization by opportunistic and pathogenic bacteria. Beyond this barrier effect, MOS interacts with GALT, stimulating immune signaling pathways that enhance mucosal immunity and improve overall gut integrity. This dual action improves mucosal immunity, gut health, and overall growth performance in aquaculture species [132]. Importantly, yeast‐derived products may also exert synergistic effects when combined with probiotics. For instance, the coadministration of *Saccharomyces cerevisiae* with lactic acid bacteria (LAB) has been reported to enhance mucosal IgT production and strengthen antiviral defenses, indicating that yeast derivatives and bacterial probiotics can play complementary roles in modulating host immunity. Such synergistic interactions show the potential of multicomponent functional feeds to provide broader protection against pathogens in aquaculture [128].

### 4.4. Algae‐Based Immunostimulants: Molecular Mechanisms and Biomarkers

Oceans cover nearly 70% of the Earth’s surface and represent an underexplored reservoir of biologically active compounds, particularly valuable as terrestrial resources become increasingly limited and overexploited. Various marine organisms, including invertebrates, algae, and microbes, are known producers of diverse natural products with applications in the pharmaceutical, cosmetic, food, and feed industries, and in recent years in aquaculture [133, 134]. Among these, algae have gained considerable attention in aquaculture due to their unique biochemical composition and broad spectrum of bioactivities.

The use of macroalgae (e.g., *Ulva*, *Laurencia*, *Sargassum*, and *Laminaria*) and microalgae (e.g., *Pediastrum*, *Spirulina*, *Chlorella*, and *Nannochloropsis*) as immunostimulants in aquaculture has gained increasing attention in recent years because of their rich composition of bioactive compounds, including polysaccharides (e.g., β‐glucans, ulvans, fucoidans, and carrageenans), polyphenolic compounds, AMPs, natural pigments such as carotenoids, essential fatty acids, vitamins, and trace elements [135].

Algae‐derived bioactives may interact with host PRRs, including TLRs and C‐type lectins, triggering downstream signaling cascades that regulate cytokine production and immune gene expression, ultimately enhancing both innate and adaptive immune responses. Dietary supplementation with *Pediastrum boryanum* in tilapia resulted in the downregulation of the proinflammatory cytokine TNF‐A, concomitant with upregulation of the anti‐inflammatory cytokines IL‐10 and TGF‐β1, indicating a balanced immunomodulatory effect [136]. Dietary inclusion of *Chlorella peruviana* has been shown to significantly enhance the innate immune competence of rainbow trout (*Oncorhynchus mykiss*) fingerlings. Supplementation increased serum lysozyme and alternative complement pathway activity (ACH50), together with elevated leukocyte and lymphocyte counts [137].

It has been reported that, in some cases, the combined use of different algal species positively affects the immune response. Sanchez et al. [138] demonstrated that feeding *Nannochloropsis gaditana* and *Schizochytrium* spp. greatly improved nonspecific innate immune responses in Atlantic salmon (*Salmo salar*) fingerlings. In erythrocytes, transcriptional upregulation was detected for critical antibacterial effectors such as complement component C3 and antibiotic peptide NK‐lysin. The quantity of circulating monocytes and immature erythrocytes has also increased hematopoietic and phagocytic activity. Ferreira et al. [139] showed that dietary supplementation of a micro‐ and macroalgae blend containing *Nannochloropsis oceanica*, *Chlorella vulgaris*, *Gracilaria gracilis*, and *Ulva rigida* modulated both mucosal and systemic immunity in European seabass (*Dicentrarchus labrax*) against *Tenacibaculum maritimum* bacteria. The algal combination diet resulted in increased monocyte and lymphocyte numbers in skin and intestinal tissues, as well as activation of genes related to cell migration and proliferation (mmp9 and pcna) and proinflammatory signaling (IL‐1B, il‐8). Plasma bactericidal activity, along with the AMP hepcidin expression, was significantly higher. Furthermore, the algae diet resulted in improved neutrophil circulation, and the increased goblet cell number led to lower pathogen colonization and mortality rates. In another study, *Arthrospira platensis* in combination with lemongrass (plant) has been shown to strengthen host defense in Nile tilapia (*Oreochromis niloticus*) by coordinating redox regulation with cytokine‐mediated immune activation, thereby improving systemic resistance to bacterial infection against *Aeromonas hydrophila* [140].

The immune response may vary not only with the species employed but also with factors such as dosage, duration of administration, and form of application. Méndez‐Vivancos et al. [141] showed that a *Nannochloropsis gaditana*‐supplemented diet in raw or hydrolyzed form modulates innate immunity differently in juvenile sea bream (*Sparus aurata*) in the medium and long term. It has been reported that microalgae feeding, regardless of format, modulates innate immunity slightly in the medium term. In the long term, however, hydrolyzed microalgae diets have been reported to be ineffective in immunomodulation, while raw microalgae diets have been associated with increased postinfection mortality rates due to excessive upregulation of the proinflammatory cytokine il6 and the AMP hepcidin. On the other hand, in gilthead seabream (*Sparus aurata*), *Laminaria digitata* inclusion did not induce chronic inflammation but instead maintained immunological homeostasis, showing that algal supplementation is safe even during long‐term feeding [142]. The immunomodulatory effect of *Arthrospira platensis* (Spirulina), a well‐characterized blue‐green alga rich in natural AMPs [143], has been reported when used as a functional feed ingredient in many species [144], such as Nile tilapia (*Oreochromis niloticus*) [145], rainbow trout (*Oncorhynchus mykiss*) [146], and stinging catfish (*Heteropneustes fossilis*) [147].

## 5. Future Perspectives

Aquaculture has been projected to sustainably bridge the gap between the demand and supply of aquatic dietary proteins by 2050. It is therefore mandatory that novel policies and noninvasive production strategies targeting animal welfare and public health safety must be implemented. For this, the EU has restricted the use of antibiotics in aquaculture with the aim of reducing the risk of antimicrobial resistance and strengthening the development of sustainable prophylaxis and therapeutics [148]. In this context, there is increasing research and a booming market for sustainable functional feed additives with health benefits for aquaculture [149]. While several dietary immunostimulants have been proven to be remarkably effective in promoting fish immunity, more efforts need to be channeled toward elucidating the underlying mechanisms of action of the tested functional ingredients on fish immune components. This is key for the standardized use of functional feeds and their commercial applications. Although there is limited information on the specific mechanisms, it is postulated that activation of PRRs and signal transduction pathways (NF‐KB, MAPK, and JAK/STAT) by dietary immunostimulants might be responsible for the observed immune responses and further explain the fish ability to resist diseases (Table 2).

A multiomics strategy, integrating transcriptomics, proteomics, metabolomics, and microbiome profiling, offers a systems‐level perspective on understanding the effects of functional feeds on cellular pathways and host immunity. Such approaches enable the discovery of biomarkers linking diet composition to immune competence and disease resistance [16, 17]. For example, coupling microbiome sequencing with host transcriptomics has revealed how probiotics and prebiotics reshape gut microbial communities while modulating host cytokine networks [150]. Early‐life microbiome–immune crosstalk is especially critical: exposure to microbial ligands such as β‐glucans or PGN fragments during larval development can “train” innate immunity, providing enhanced protection even before adaptive immunity matures [26, 151]. These findings underscore the potential of functional feeds not only as nutritional supplements but also as immunological training agents in aquaculture. Coupling omics datasets with artificial intelligence (AI) and machine learning may allow predictive modeling of immune responses, supporting the design of precision immunostimulant feeds tailored to specific species, developmental stages, and even farm conditions. Parallel to this, CRISPR‐based functional studies could be applied to validate molecular targets such as PRRs, thereby clarifying the precise signaling pathways triggered by feed‐derived bioactives.

Another promising field is microbiome engineering, where functional feeds are formulated to shape gut microbial communities into stable, disease‐resilient ecosystems. Beyond single probiotics, the use of synthetic microbial consortia or postbiotics may provide long‐term benefits for host immunity and stress tolerance. Equally, the valorization of agro‐industrial and fishery by‐products (e.g., seaweed residues, shellfish exoskeletons, and yeast biomass) as sources of immunostimulants fits well within a circular bioeconomy, aligning aquaculture with sustainability goals.

Emerging technologies like nanomaterial‐based delivery systems, such as chitosan and ulvan‐loaded nanoparticles (CS‐UL‐TPP NPs), offer the potential to enhance the bioavailability and immunostimulatory effects of functional feed additives. A study by [152] recommended CS‐UL‐TPP NPs as immunostimulants in feed and showed antibacterial activity against *P. damselae* subsp. *piscicida* and stimulated immune responses, including increased lysozyme activity and upregulation of immune genes after oral administration of CS‐UL‐TPP NPs in Senegalese sole (*Solea senegalensis* Kaup, 1858) juveniles [152]. Future efforts may focus on developing smart, responsive feed particles that release immunostimulants only when triggered by specific physiological or environmental cues. For example, encapsulating immunostimulants within micro‐ or nanoparticles engineered to react to gut pH, temperature, or the presence of pathogens could greatly enhance targeted immune activation while reducing waste and cost. Such hybrid systems could synergize with probiotics, enzymes, or bioactive peptides, pushing the boundaries of sustainable aquafeed innovation.

## 6. Concluding Remarks

Functional feeds represent one of the most promising innovations for sustainable aquaculture, offering environmentally friendly alternatives to antibiotics and chemotherapeutics. By harnessing nutrient‐, plant‐, microbial‐, and algae‐based immunostimulants, aquaculture can improve fish health, enhance disease resistance, and support food security. However, their successful application requires deeper mechanistic insights, integration of advanced molecular tools, and the development of precision formulations adapted to the species and farming conditions. The convergence of multiomics, AI‐driven modeling, microbiome research, and nanotechnology will likely define the next generation of functional feeds. Coupled with sustainability‐driven policies and regulatory frameworks, these advances can transform functional feeds from promising concepts into reliable cornerstones of global aquaculture health management.

In conclusion, functional feeds represent a promising strategy for modulating immune responses in aquaculture species, and molecular biomarkers provide valuable insights into the underlying mechanisms of immunostimulation. However, it must be acknowledged that molecular indicators alone are insufficient to fully characterize the immune status of aquatic animals. A scientifically robust assessment of immune competence requires the integration of molecular data with biochemical, cellular, and physiological indicators. Such integrative strategies will enhance the reliability of immune assessments and improve the development and validation of functional feeds as immunostimulants in sustainable aquaculture.

## Author Contributions

**Uzeme Precious Aluta**: conceptualization. All authors contributed to data curation, visualization, and writing – original draft, review, and editing.

## Acknowledgments

The authors appreciate Prof. Margaret Crumlish for reviewing the manuscript for scientific english, grammatical, and typographical errors.

## Funding

This article is based on work from the BioAqua COST Action (CA22160) and supported by COST (European Cooperation in Science and Technology).

## Ethics Statement

Ethical approval and informed consent were not required, as this is a review based exclusively on previously published articles.

## Conflicts of Interest

The authors declare no conflicts of interest.

## Data Availability

This article is a review and does not contain any original data. All data discussed are from previously published studies, which are cited within the manuscript.

## References

[1] FAO , FAO Report: Global Fisheries and Aquaculture Production Reaches a New Record High, 2024, Newsroom.

[2] Ruenkoed S. , Nontasan S. , and Phudkliang J. , et al.Effect of Dietary Gamma Aminobutyric Acid (GABA) Modulated the Growth Performance, Immune and Antioxidant Capacity, Digestive Enzymes, Intestinal Histology and Gene Expression of Nile Tilapia (*Oreochromis niloticus*), Fish & Shellfish Immunology. (2023) 141, 10.1016/j.fsi.2023.109056, 109056. 37673386

[3] Costello C. , Cao L. , and Gelcich S. , et al.The Future of Food From the Sea, Nature. (2020) 588, no. 7836, 95–100, 10.1038/s41586-020-2616-y. 32814903

[4] Maezono M. , Nielsen R. , Buchmann K. , and Nielsen M. , The Current State of Knowledge of the Economic Impact of Diseases in Global Aquaculture, Reviews in Aquaculture. (2025) 17, no. 3.

[5] Lafferty K. D. , Harvell C. D. , and Conrad J. M. , et al.Infectious Diseases Affect Marine Fisheries and Aquaculture Economics, Annual Review of Marine Science. (2015) 7, no. 1, 471–496. 10.1146/annurev-marine-010814-015646 25251276

[6] Gupta A. , Gupta S. K. , and Priyam M. , et al.Immunomodulation by Dietary Supplements: A Preventive Health Strategy for Sustainable Aquaculture of Tropical Freshwater Fish, *Labeo rohita* (Hamilton, 1822), Reviews in Aquaculture. (2021) 13, no. 4, 2364–2394, 10.1111/raq.12581.

[7] Kumar H. , Kawai T. , and Akira S. , Pathogen Recognition by the Innate Immune System, International Reviews of Immunology. (2011) 30, no. 1, 16–34, 10.3109/08830185.2010.529976. 21235323

[8] Callol A. , Roher N. , Amaro C. , and MacKenzie S. , Characterization of PAMP/PRR Interactions in European eel (*Anguilla anguilla*) Macrophage-Like Primary Cell Cultures, Fish & Shellfish Immunology. (2013) 35, no. 4, 1216–1223, 10.1016/j.fsi.2013.07.037. 23911651

[9] Misra C. K. , Das B. K. , Mukherjee S. C. , and Pattnaik P. , Effect of Long-Term Administration of Dietary β-Glucan on Immunity, Growth and Survival of *Labeo rohita* Fingerlings, Aquaculture. (2006) 255, no. 1–4, 82–94, 10.1016/j.aquaculture.2005.12.009.

[10] Biswas G. , Korenaga H. , Takayama H. , Kono T. , Shimokawa H. , and Sakai M. , Cytokine Responses in the Common Carp, *Cyprinus carpio*, L. Treated With Baker’s Yeast Extract, Aquaculture. (2012) 356-357, 169–175, 10.1016/j.aquaculture.2012.05.019.

[11] Falco Gracia J. A. , Miest J. J. , and Pionnier N. , et al.B-Glucan-Supplemented Diets Increase Poly (I: C)-Induced Gene Expression of Mx, Possibly via Tlr3-Mediated Recognition Mechanism in Common Carp (*Cyprinus carpio*), Fish & Shellfish Immunology. (2014) 36, no. 2, 494–502, 10.1016/j.fsi.2013.12.005. 24370748

[12] Xu T. , Wang J. , and Xu H. , et al.Dietary β-1, 3-Glucan Promotes Growth Performance and Enhances Non-Specific Immunity by Modulating Pattern Recognition Receptors in Juvenile Oriental River Prawn (*Macrobrachium nipponense*), Fishes. (2024) 9, no. 10, 10.3390/fishes9100379, 379.

[13] Marcharla E. , Vishnuprasadh A. , Gnanasekaran L. , Vinayagam S. , Sundaram T. , and Ganesan S. , The Role of Functional Feed in Modulating Fish Gut Microbiome to Enhance Resistance Against Aquaculture Pathogens, Probiotics and Antimicrobial Proteins. (2026) 18, no. 2, 3010–3039, 10.1007/s12602-025-10660-w. 40694305

[14] Smith N. C. , Rise M. L. , and Christian S. L. , A Comparison of the Innate and Adaptive Immune Systems in Cartilaginous Fish, Ray-Finned Fish, and Lobe-Finned Fish, Frontiers in Immunology. (2019) 10, 10.3389/fimmu.2019.02292, 2292. 31649660 PMC6795676

[15] Natnan M. E. , Low C.-F. , Chong C.-M. , Bunawan H. , and Baharum S. N. , Integration of Omics Tools for Understanding the Fish Immune Response Due to Microbial Challenge, Frontiers in Marine Science. (2021) 8, 10.3389/fmars.2021.668771, 668771.

[16] Martin S. A. and Król E. , Nutrigenomics and Immune Function in Fish: New Insights From Omics Technologies, Developmental & Comparative Immunology. (2017) 75, 86–98, 10.1016/j.dci.2017.02.024. 28254621 PMC5495911

[17] Porter D. , Peggs D. , McGurk C. , and Martin S. A. M. , Immune Responses to Prebiotics in Farmed Salmonid Fish: How Transcriptomic Approaches Help Interpret Responses, Fish & Shellfish Immunology. (2022) 127, 35–47, 10.1016/j.fsi.2022.05.055. 35667538

[18] Agboola J. O. , Øverland M. , Skrede A. , and Hansen J.Ø. , Yeast as Major Protein-Rich Ingredient in Aquafeeds: A Review of the Implications for Aquaculture Production, Reviews in Aquaculture. (2021) 13, no. 2, 949–970, 10.1111/raq.12507.

[19] Ringø E. , Hoseinifar S. H. , Van Doan H. , and Zhou Z. , Probiotics in Aquaculture: Trends and Future Perspectives, Reviews in Aquaculture. (2024) 16, no. 3, 1333–1350, 10.1111/raq.12899.

[20] Yusuf J. , Ahmad P. Z. , Rather M. A. , and Ahmad I. , An Overview of Functional Genomics in Aquaculture, 2025, Enhancing Food Security and Nutrition, 273–286.

[21] Shi Y. , Kong W. , and Gong F. , et al.Yeast β-Glucan Enhances the Intestinal Immune Function in Coho Salmon via the Modulation of Gut Microbiota-Mediated Lipid Metabolism, Aquaculture. (2025) 599, 10.1016/j.aquaculture.2025.742123, 742123.

[22] Sultana S. , Biró J. , Kucska B. , and Hancz C. , Factors Affecting Yeast Digestibility and Immunostimulation in Aquatic Animals, Animals. (2024) 14, no. 19, 10.3390/ani14192851, 2851. 39409800 PMC11475639

[23] Calduch-Giner J. A. , Sitjà-Bobadilla A. , and Pérez-Sánchez J. , Gene Expression Profiling Reveals Functional Specialization Along the Intestinal Tract of a Carnivorous Teleostean Fish (*Dicentrarchus labrax*), Frontiers in Physiology. (2016) 7, 10.3389/fphys.2016.00359, 359. 27610085 PMC4997091

[24] Ngoh S. Y. , Tan D. , and Shen X. , et al.Nutrigenomic and Nutritional Analyses Reveal the Effects of Pelleted Feeds on Asian Seabass (*Lates calcarifer*), PLoS ONE. (2015) 10, no. 12, 10.1371/journal.pone.0145456, e0145456. 26696533 PMC4687856

[25] García-Angulo A. , Merlo M. A. , Rodríguez M. E. , Portela-Bens S. , Liehr T. , and Rebordinos L. , Genome and Phylogenetic Analysis of Genes Involved in the Immune System of *Solea senegalensis*–Potential Applications in Aquaculture, Frontiers in Genetics. (2019) 10, 10.3389/fgene.2019.00529, 529. 31244883 PMC6579814

[26] Auclert L. Z. , Chhanda M. S. , and Derome N. , Interwoven Processes in Fish Development: Microbial Community Succession and Immune Maturation, PeerJ. (2024) 12, 10.7717/peerj.17051, e17051. 38560465 PMC10981415

[27] Núñez-Acuña G. , Détrée C. , Gallardo-Escárate C. , and Gonçalves A. T. , Functional Diets Modulate lncRNA-Coding RNAs and Gene Interactions in the Intestine of Rainbow Trout *Oncorhynchus mykiss* , Marine Biotechnology. (2017) 19, no. 3, 287–300, 10.1007/s10126-017-9750-z. 28500613

[28] Martinez-Rubio L. , Evensen Ø. , and Krasnov A. , et al.Effects of Functional Feeds on the Lipid Composition, Transcriptomic Responses and Pathology in Heart of Atlantic Salmon (*Salmo salar* L.) Before and After Experimental Challenge With Piscine Myocarditis Virus (PMCV), BMC Genomics. (2014) 15, 462. 24919788 10.1186/1471-2164-15-462 PMC4079957

[29] Vallejos Vidal E. C. , Molecular Regulation of the Immune Function in the Gills of Gilthead Sea Bream (*sparus aurata*) Fed With Immunostimulant Diets; Universitat Autònoma De, 2015, Universitat Autònoma de Barcelona.

[30] Ghaedi G. , Keyvanshokooh S. , Azarm H. M. , and Akhlaghi M. , Proteomic Analysis of Muscle Tissue From Rainbow Trout (*Oncorhynchus mykiss*) Fed Dietary β-Glucan, Iranian Journal of Veterinary Research. (2016) 17, no. 3, 184–189. 27822248 PMC5090152

[31] Lulijwa R. , Alfaro A. C. , and Young T. , Metabolomics in Salmonid Aquaculture Research: Applications and Future Perspectives, Reviews in Aquaculture. (2022) 14, no. 2, 547–577, 10.1111/raq.12612.

[32] Kurata S. , Peptidoglycan Recognition Proteins in Drosophila Immunity, Developmental & Comparative Immunology. (2014) 42, no. 1, 36–41, 10.1016/j.dci.2013.06.006. 23796791 PMC3808481

[33] Zhang J. , Li M. , and Meng D. , et al.Review on Omics Approaches in Aquatic Animal Nutrition: Current Status, Limitations, and Perspectives, The Journal of Nutrition. (2025) 155, no. 10, 3191–3210, 10.1016/j.tjnut.2025.08.019. 40818745

[34] Elumalai P. , Soltani M. , and Lakshmi S. , Immunomodulators in Aquaculture and Fish Health, 2023, CRC Press.

[35] Rocha S. D. , Valenzuela C. A. , and Morales-Lange B. , Immunonutrition 14;Contributing to the Future of Sustainable Aquaculture by Supporting Animal Performance, Health and Welfare, Animals. (2024) 14, no. 15, 10.3390/ani14152275, 2275. 39123801 PMC11311005

[36] Teixeira C. , Peixoto D. , and Hinzmann M. , et al.Dietary Strategies to Modulate the Health Condition and Immune Responses in Gilthead Seabream (*Sparus aurata*) Juveniles Following Intestinal Inflammation, Animals. (2022) 12, no. 21, 10.3390/ani12213019, 3019. 36359143 PMC9657010

[37] Zhang Z. , Chi H. , and Dalmo R. A. , Trained Innate Immunity of Fish Is a Viable Approach in Larval Aquaculture, Frontiers in Immunology. (2019) 10, 10.3389/fimmu.2019.00042, 42. 30740103 PMC6355669

[38] Hopo M. G. , Mabrok M. , Abu-Elala N. , and Yu Y. , Navigating Fish Immunity: Focus on Mucosal Immunity and the Evolving Landscape of Mucosal Vaccines, Biology. (2024) 13, no. 12, 10.3390/biology13120980, 980. 39765647 PMC11727089

[39] Aghamohammad S. , Sepehr A. , Miri S. T. , Najafi S. , Rohani M. , and Pourshafiea M. R. , The Effects of the Probiotic Cocktail on Modulation of the NF-kB and JAK/STAT Signaling Pathways Involved in the Inflammatory Response in Bowel Disease Model, BMC Immunology. (2022) 23, no. 1, 10.1186/s12865-022-00484-6, 8. 35240996 PMC8896082

[40] Lall S. P. and Kaushik S. J. , Nutrition and Metabolism of Minerals in Fish, Animals. (2021) 11, no. 9, 10.3390/ani11092711, 2711. 34573676 PMC8466162

[41] Prabhu P. A. J. , Schrama J. W. , and Kaushik S. J. , Mineral Requirements of Fish: A Systematic Review, Reviews in Aquaculture. (2016) 8, no. 2, 172–219.

[42] Won S. , Moniruzzaman M. , and Lee S. , et al.Evaluation of Dietary Natural Mineral Materials as an Antibiotic Replacer on Growth Performance, Non-Specific Immune Responses and Disease Resistance in Rainbow Trout, *Oncorhynchus mykiss* , Aquaculture Research. (2017) 48, no. 9, 4735–4747, 10.1111/are.13295.

[43] Yu H. , Mushtaq M. , and Razzaq S. , et al.Effects of Dietary Organic Selenium, Vitamin C, and Vitamin E Supplementation on Growth Performance, Serum Biochemistry, and Antioxidant Status in Juvenile *Hypophthalmichthys molitrix* , Frontiers in Marine Science. (2025) 12, 10.3389/fmars.2025.1555994, 1555994.

[44] Rathore S. S. , Hanumappa S. M. , and Yusufzai S. I. , et al.Dietary Administration of Engineered Nano-Selenium and Vitamin C Ameliorates Immune Response, Nutritional Physiology, Oxidative Stress, and Resistance Against *Aeromonas hydrophila* in Nile Tilapia (Oreochromis niloticus), Biological Trace Element Research. (2023) 201, no. 8, 4079–4092, 10.1007/s12011-022-03473-3. 36374364

[45] Gil A. , Modulation of the Immune Response Mediated by Dietary Nucleotides, European Journal of Clinical Nutrition. (2002) 56, no. S3, S1–S4, 10.1038/sj.ejcn.1601475. 12142952

[46] Gatlin D. M. and Li P. , Nakagawa H. , Sato M. , and Gatlin D. M.III , Nucleotides, Dietary Supplements for the Health and Quality of Cultured Fish, 2007, CABI, 193–209.

[47] Hossain M. S. , Koshio S. , and Kestemont P. , Recent Advances of Nucleotide Nutrition Research in Aquaculture: A Review, Reviews in Aquaculture. (2020) 12, no. 2, 1028–1053, 10.1111/raq.12370.

[48] Bae J. , Song Y. , and Moniruzzaman M. , et al.Evaluation of Dietary Soluble Extract Hydrolysates With or Without Supplementation of Inosine Monophosphate Based on Growth, Hematology, Non-Specific Immune Responses and Disease Resistance in Juvenile Nile Tilapia *Oreochromis niloticus* , Animals. (2021) 11, no. 4, 10.3390/ani11041107, 1107. 33921530 PMC8070139

[49] Choi W. , Moniruzzaman M. , and Bae J. , et al.Evaluation of Dietary Probiotic Bacteria and Processed Yeast (GroPro-Aqua) as the Alternative of Antibiotics in Juvenile Olive Flounder *Paralichthys olivaceus* , Antibiotics. (2022) 11, no. 2, 10.3390/antibiotics11020129, 129. 35203732 PMC8868502

[50] Rashmeei M. , Shekarabi S. P. H. , Mehrgan M. S. , and Paknejad H. , Stimulatory Effect of Dietary Chasteberry (*Vitex agnus-castus*) Extract on Immunity, Some Immune-Related Gene Expression, and Resistance Against *Aeromonas hydrophila* Infection in Goldfish (*Carassius auratus*), Fish & Shellfish Immunology. (2020) 107, 129–136, 10.1016/j.fsi.2020.09.037. 33002603

[51] Salomón R. , Firmino J. P. , and Reyes-López F. E. , et al.The Growth Promoting and Immunomodulatory Effects of a Medicinal Plant Leaf Extract Obtained From *Salvia officinalis* and *Lippia citriodora* in Gilthead Seabream (*Sparus aurata*), Aquaculture. (2020) 524, 10.1016/j.aquaculture.2020.735291, 735291.

[52] Reyes-Becerril M. , Maldonado M. , Vimolmangkang S. , and Angulo C. , In Vivo and Ex Vivo Studies Support the Immunostimulant and Immunoprotective Effect of Damiana (*Turnera diffusa* Willd) in Almaco Jack (*Seriola rivoliana*), Fish & Shellfish Immunology. (2024) 146, 10.1016/j.fsi.2024.109369, 109369. 38220122

[53] Tan X. and Sun Z. , Dietary Dandelion Extract Improved Growth Performance, Immunity, Intestinal Morphology and Microbiota Composition of Golden Pompano *Trachinotus ovatus* , Aquaculture Reports. (2020) 18, 10.1016/j.aqrep.2020.100491, 100491.

[54] Bilen S. , Mohamed Ali G. A. , Amhamed I. D. , and Almabrok A. A. , Modulatory Effects of Laurel-Leaf Cistus (*Cistus laurifolius*) Ethanolic Extract on Innate Immune Responses and Disease Resistance in Common Carp (*Cyprinus carpio*), Fish & Shellfish Immunology. (2021) 116, 98–106, 10.1016/j.fsi.2021.07.001. 34252543

[55] Yilmaz S. , Ergün S. , Yilmaz E. , Ahmadifar E. , Yousefi M. , and Abdel-Latif H. M. , Effects of a Phytogenic Diet on Growth, Haemato-Immunological Parameters, Expression of Immune-and Stress-Related Genes, and Resistance of *Oncorhynchus mykiss* to *Lactococcus garvieae* Infection, Aquaculture. (2024) 587, 10.1016/j.aquaculture.2024.740845, 740845.

[56] Armobin K. , Ahmadifar E. , and Adineh H. , et al.Quercetin Application for Common Carp (*Cyprinus carpio*): I. Effects on Growth Performance, Humoral Immunity, Antioxidant Status, Immune-Related Genes, and Resistance Against Heat Stress, Aquaculture Nutrition. (2023) 2023, no. 1, 10.1155/2023/1168262, 1168262. 36860974 PMC9973228

[57] Abdel-Latif H. M. , Hendam B. M. , Nofal M. I. , and El-Son M. A. , *Ginkgo biloba* Leaf Extract Improves Growth, Intestinal Histomorphometry, Immunity, Antioxidant Status, and Modulates Transcription of Cytokine Genes in Hapa-Reared *Oreochromis niloticus* , Fish & Shellfish Immunology. (2021) 117, 339–349, 10.1016/j.fsi.2021.06.003. 34153429

[58] Adel M. , Dawood M. A. , Shafiei S. , Sakhaie F. , and Shekarabi S. P. H. , Dietary *Polygonum minus* Extract Ameliorated the Growth Performance, Humoral Immune Parameters, Immune-Related Gene Expression, and Resistance Against *Yersinia ruckeri* in Rainbow Trout (*Oncorhynchus mykiss*), Aquaculture. (2020) 519, 10.1016/j.aquaculture.2019.734738, 734738.

[59] Baba E. , Acar Ü. , Yılmaz S. , Zemheri F. , and Ergün S. , Dietary Olive Leaf (*Olea Europea* L.) Extract Alters Some Immune Gene Expression Levels and Disease Resistance to *Yersinia ruckeri* Infection in Rainbow Trout *Oncorhynchus mykiss* , Fish & Shellfish Immunology. (2018) 79, 28–33, 10.1016/j.fsi.2018.04.063. 29733961

[60] Chaklader M. R. , Ahmed H. A. , Khafaga A. F. , Shukry M. , Selema T. A. A. , and Abdel-Latif H. M. , *Silybum marianum* Promotes Growth, Hepatic Antioxidative Activity, and Splenic Immunity but Does Not Influence the Intestinal Barrier Function of Nile Tilapia, *Oreochromis niloticus* , Aquaculture. (2024) 583, 10.1016/j.aquaculture.2024.740554, 740554.

[61] Hendam B. M. , Baromh M. Z. , Khafaga A. F. , Shukry M. , El-Son M. A. , and Abdel-Latif H. M. , Effects of Dietary Baobab, *Adansonia digitata* on Growth, Haemato-Immunological Status, Antioxidant Biomarkers, Intestinal Histomorphometry, Gene Expression Responses, and Disease Resistance in Nile tilapia, *Oreochromis niloticus* , Aquaculture. (2024) 581, 10.1016/j.aquaculture.2023.740473, 740473.

[62] Mehrabi Z. , Firouzbakhsh F. , Rahimi-Mianji G. , and Paknejad H. , Immunostimulatory Effect of *Aloe vera* (*Aloe barbadensis*) on Non-Specific Immune Response, Immune Gene Expression, and Experimental Challenge With *Saprolegnia parasitica* in Rainbow Trout (*Oncorhynchus mykiss*), Aquaculture. (2019) 503, 330–338, 10.1016/j.aquaculture.2019.01.025.

[63] Shekarabi S. P. H. , Mehrgan M. S. , and Ramezani F. , et al.Effect of Dietary Barberry Fruit (*Berberis vulgaris*) Extract on Immune Function, Antioxidant Capacity, Antibacterial Activity, and Stress-Related Gene Expression of Siberian Sturgeon (*Acipenser baerii*), Aquaculture Reports. (2022) 23, 10.1016/j.aqrep.2022.101041, 101041.

[64] Yilmaz E. , Motlagh H. A. , and Yilmaz S. , Impact of Valerian (*Valeriana officinalis*) Extract Supplementation on Common Carp (*Cyprinus carpio* L.) Growth Performance, Immune Responses, and Gene Expression, Aquaculture Research. (2025) 2025, no. 1, 7950238.

[65] Foysal M. J. , Alam M. , and Momtaz F. , et al.Dietary Supplementation of Garlic (*Allium sativum*) Modulates Gut Microbiota and Health Status of Tilapia (*Oreochromis niloticus*) Against *Streptococcus iniae* Infection, Aquaculture Research. (2019) 50, no. 8, 2107–2116, 10.1111/are.14088.

[66] Giri S. S. , Kim S. G. , and Jung W. J. , et al.Dietary *Syzygium cumini* Leaf Extract Influences Growth Performance, Immunological Responses and Gene Expression in Pathogen-Challenged *Cyprinus carpio* , Fish & Shellfish Immunology. (2023) 138, 10.1016/j.fsi.2023.108830, 108830. 37244318

[67] Salomón R. , Furones M. D. , and Reyes-López F. E. , et al.A Bioactive Extract Rich in Triterpenic Acid and Polyphenols From Olea Europaea Promotes Systemic Immunity and Protects Atlantic Salmon Smolts Against Furunculosis, Frontiers in Immunology. (2021) 12, 10.3389/fimmu.2021.737601, 737601. 34867959 PMC8633542

[68] Paray B. A. , Hoseini S. M. , Hoseinifar S. H. , and Van Doan H. , Effects of Dietary Oak (*Quercus castaneifolia*) Leaf Extract on Growth, Antioxidant, and Immune Characteristics and Responses to Crowding Stress in Common Carp (*Cyprinus carpio*), Aquaculture. (2020) 524, 10.1016/j.aquaculture.2020.735276, 735276.

[69] Assar D. H. , Ragab A. E. , and Abdelsatar E. , et al.Dietary Olive Leaf Extract Differentially Modulates Antioxidant Defense of Normal and *Aeromonas hydrophila*-Infected Common Carp (*Cyprinus carpio*) via Keap1/Nrf2 Pathway Signaling: A Phytochemical and Biological Link, Animals. (2023) 13, no. 13, 10.3390/ani13132229, 2229. 37444027 PMC10339993

[70] Monod E. C. , Betancourt J. L. , and Samms K. A. , et al.Immunostimulant Effects of Pituitary Adenylate Cyclase-Activating Polypeptide and Double-Stranded (ds) RNA in *Orconectes propinquus* , Fish & Shellfish Immunology. (2024) 146, 10.1016/j.fsi.2024.109388, 109388. 38244819

[71] Karataş B. , Dietary *Cyanus depressus* (M. Bieb.) Soják Plant Extract Enhances Growth Performance, Modulates Intestinal Microbiota, and Alters Gene Expression Associated With Digestion, Antioxidant, Stress, and Immune Responses in Rainbow Trout (*Oncorhynchus mykiss*), Aquaculture International. (2024) 32, no. 6, 7929–7951, 10.1007/s10499-024-01548-7.

[72] Reyes-Cerpa S. , Vallejos-Vidal E. , and Gonzalez-Bown M. J. , et al.Effect of Yeast (*Xanthophyllomyces dendrorhous*) and Plant (Saint John’s Wort, Lemon Balm, and Rosemary) Extract Based Functional Diets on Antioxidant and Immune Status of Atlantic Salmon (*Salmo salar*) Subjected to Crowding Stress, Fish & Shellfish Immunology. (2018) 74, 250–259, 10.1016/j.fsi.2017.12.061. 29305990

[73] Darvishi M. , Shamsaie Mehrgan M. , and Khajehrahimi A. E. , Effect of Licorice (*Glycyrrhiza glabra*) Extract as an Immunostimulant on Serum and Skin Mucus Immune Parameters, Transcriptomic Responses of Immune-Related Gene, and Disease Resistance Against *Yersinia ruckeri* in Rainbow Trout (*Oncorhynchus mykiss*), Frontiers in Veterinary Science. (2022) 9, 10.3389/fvets.2022.811684, 811684. 35280148 PMC8904569

[74] Shehata A. I. , Taha S. A. , and Elmaghraby A. M. , et al.Effects of Dietary Bay Leaf (*Laurus nobilis*) Aqueous Extract on Growth Performance, Feed Utilization, Antioxidant Activity, Immunity, and Gene Expression in Nile Tilapia (*Oreochromis niloticus*), Aquaculture. (2025) 599, 10.1016/j.aquaculture.2025.742155, 742155.

[75] Firmino J. P. , Galindo-Villegas J. , Reyes-López F. E. , and Gisbert E. , Phytogenic Bioactive Compounds Shape Fish Mucosal Immunity, Frontiers in Immunology. (2021) 12, 10.3389/fimmu.2021.695973, 695973. 34220858 PMC8252966

[76] Vallejos-Vidal E. , Reyes-López F. , Teles M. , and MacKenzie S. , The Response of Fish to Immunostimulant Diets, Fish & Shellfish Immunology. (2016) 56, 34–69, 10.1016/j.fsi.2016.06.028. 27389620

[77] Somensi N. , Rabelo T. K. , and Guimarães A. G. , et al.Carvacrol Suppresses LPS-Induced Pro-Inflammatory Activation in RAW. 264.7 Macrophages Through ERK1/2 and NF-kB Pathway, International Immunopharmacology. (2019) 75, 10.1016/j.intimp.2019.105743, 105743. 31357087

[78] Elumalai P. , Kurian A. , Lakshmi S. , Faggio C. , Esteban M. A. , and Ringø E. , Herbal Immunomodulators in Aquaculture, Reviews in Fisheries Science & Aquaculture. (2021) 29, no. 1, 33–57, 10.1080/23308249.2020.1779651.

[79] Jia R. , Gu Z. , and He Q. , et al.Anti-Oxidative, Anti-Inflammatory and Hepatoprotective Effects of *Radix Bupleuri* Extract Against Oxidative Damage in Tilapia (*Oreochromis niloticus*) via Nrf2 and TLRs Signaling Pathway, Fish & Shellfish Immunology. (2019) 93, 395–405, 10.1016/j.fsi.2019.07.080. 31374313

[80] Vijayaram S. , Sun Y. Z. , Zuorro A. , Ghafarifarsani H. , Van Doan H. , and Hoseinifar S. H. , Bioactive Immunostimulants as Health-Promoting Feed Additives in Aquaculture: A Review, Fish & Shellfish Immunology. (2022) 130, 294–308, 10.1016/j.fsi.2022.09.011. 36100067

[81] Mensah D. D. , Montero R. , Morales-Lange B. , Øverland M. , and Mydland L. T. , In Vitro Salmonid Models as Tools for Studying Microbial-Derived Immunostimulants and Aquaculture Relevant Salmonids Pathogens: Current Status and Future Perspectives, Aquaculture. (2025) 595, 10.1016/j.aquaculture.2024.741695, 741695.

[82] Ulvestad J. S. , Kumari J. , Seternes T. , Chi H. , and Dalmo R. A. , Studies on the Effects of LPS, ß-Glucan and Metabolic Inhibitors on the Respiratory Burst and Gene Expression in Atlantic Salmon Macrophages, Journal of Fish Diseases. (2018) 41, no. 7, 1117–1127. 29600522 10.1111/jfd.12806

[83] Samms K. A. , Alkie T. N. , Jenik K. , de Jong J. , Klinger K. M. , and DeWitte-Orr S. J. , Oral Delivery of a DsRNA-Phytoglycogen Nanoparticle Complex Enhances Both Local and Systemic Innate Immune Responses in Rainbow Trout, Fish & Shellfish Immunology. (2022) 121, 215–222, 10.1016/j.fsi.2021.12.038. 34999226

[84] Hoseinifar S. H. , Faheem M. , Liaqat I. , Van Doan H. , Ghosh K. , and Ringø E. , Promising Probiotic Candidates for Sustainable Aquaculture: An Updated Review, Animals. (2024) 14, no. 24, 10.3390/ani14243644, 3644. 39765548 PMC11672650

[85] Nguyen T. M. , Thi T. H. V. , Van S. V. , Hich T. V. , and Le H. V. , Influence of a Peptidoglycan-Enriched Diet on Growth Performance, Feed Utilization and Immune Response in Striped Catfish (*Pangasianodon hypophthalmus*), Fisheries and Aquatic Sciences. (2024) 27, no. 9, 572–579, 10.47853/FAS.2024.e54.

[86] Srirengaraj V. , Razafindralambo H. L. , Rabetafika H. N. , Nguyen H. T. , and Sun Y. Z. , Sustainable Biotic Agents in Aquaculture: Concepts, 2023, Action Mechanisms and Applications. 10.3390/biology12121498 PMC10740583 38132324

[87] Fosse M. M. , Méndez L. R. , Rodríguez-Ramos T. , Dixon B. , Sundh H. , and Olsen R. E. , Functional Feed Ingredients Modulate the Immune Response of RTgutGC Cells to LPS-Induced Inflammation, Frontiers in Immunology. (2025) 16, 10.3389/fimmu.2025.1616076, 1616076. 40607398 PMC12219272

[88] Gupta S. K. , Sarkar B. , and Priyam M. , et al.Inflammatory and Stress Biomarker Response of *Aeromonas hydrophila* Infected Rohu, *Labeo rohita* Fingerlings to Dietary Microbial Levan, Aquaculture. (2020) 521, 10.1016/j.aquaculture.2020.735020, 735020.

[89] Wang J. , Wilson A. E. , Su B. , and Dunham R. A. , Functionality of Dietary Antimicrobial Peptides in Aquatic Animal Health: Multiple Meta-Analyses, Animal Nutrition. (2023) 12, 200–214, 10.1016/j.aninu.2022.10.001. 36712402 PMC9860427

[90] Salah A. S. , El Nahas A. F. , and Mahmoud S. , Modulatory Effect of Different Doses of β-1, 3/1, 6-Glucan on the Expression of Antioxidant, Inflammatory, Stress and Immune-Related Genes of *Oreochromis niloticus* Challenged With *Streptococcus iniae* , Fish & Shellfish Immunology. (2017) 70, 204–213, 10.1016/j.fsi.2017.09.008. 28882806

[91] Ding Z. , Wang X. , and Liu Y. , et al.Dietary Mannan Oligosaccharides Enhance the Non-Specific Immunity, Intestinal Health, and Resistance Capacity of Juvenile Blunt Snout Bream (*Megalobrama amblycephala*) Against *Aeromonas hydrophila* , Frontiers in Immunology. (2022) 13, 10.3389/fimmu.2022.863657, 863657. 35784342 PMC9240629

[92] Merrifield D. L. , Dimitroglou A. , and Foey A. , et al.The Current Status and Future Focus of Probiotic and Prebiotic Applications for Salmonids, Aquaculture. (2010) 302, no. 1-2, 1–18, 10.1016/j.aquaculture.2010.02.007.

[93] Rehman S. , Gora A. H. , and Abdelhafiz Y. , et al.Potential of Algae-Derived Alginate Oligosaccharides and β-Glucan to Counter Inflammation in Adult Zebrafish Intestine, Frontiers in Immunology. (2023) 14, 10.3389/fimmu.2023.1183701, 1183701. 37275890 PMC10235609

[94] Hoseinifar S. H. , Sun Y. Z. , Wang A. , and Zhou Z. , Probiotics as Means of Diseases Control in Aquaculture, A Review of Current Knowledge and Future Perspectives, Frontiers in Microbiology. (2018) 9, 2429. 30369918 10.3389/fmicb.2018.02429 PMC6194580

[95] Van Doan H. , Hoseinifar S. H. , Ringø E. , and Esteban M.Á. , Postbiotics and Their Applications in Aquaculture: A Review, Fish & Shellfish Immunology. (2023) 137, 10.1016/j.fsi.2023.108738, 108738. 37031922

[96] Ringø E. , Hoseinifar S. H. , Ghosh K. , Van Doan H. , Beck B. R. , and Song S. K. , Probiotics, Lactic Acid Bacteria and Bacilli: Interesting Supplementation for Aquaculture, Journal of Applied Microbiology. (2018) 124, no. 6, 1571–1586. 10.1111/jam.14628 32141152

[97] Lazado C. C. and Caipang C. M. A. , Mucosal Immunity and Probiotics in Fish, Fish & Shellfish Immunology. (2014) 39, no. 1, 78–89, 10.1016/j.fsi.2014.04.015. 24795079

[98] Porter D. , Naseer S. , Peggs D. , McGurk C. , and Martin S. A. M. , Deciphering the Immunostimulatory Effects of β-Glucan on a Rainbow Trout (*Oncorhynchus mykiss*) Macrophage-Like Cell Line (RTS11) by Whole Transcriptome Analysis, Genes. (2023) 14, no. 6, 10.3390/genes14061261, 1261. 37372441 PMC10298332

[99] Ringø E. , Van Doan H. , and Lee S. H. , et al.Probiotics, Lactic Acid Bacteria and Bacilli: Interesting Supplementation for Aquaculture, Journal of Applied Microbiology. (2020) 129, no. 1, 116–136, 10.1111/jam.14628. 32141152

[100] Selim K. M. and Reda R. M. , Improvement of Immunity and Disease Resistance in the Nile Tilapia, *Oreochromis niloticus*, by Dietary Supplementation With *Bacillus amyloliquefaciens* , Fish & Shellfish Immunology. (2015) 44, no. 2, 496–503, 10.1016/j.fsi.2015.03.004. 25783002

[101] Newaj-Fyzul A. and Austin B. , Probiotics, Immunostimulants, Plant Products and Oral Vaccines, and Their Role as Feed Supplements in the Control of Bacterial Fish Diseases, Journal of Fish Diseases. (2015) 38, no. 11, 937–955, 10.1111/jfd.12313. 25287254

[102] Fachri M. , Amoah K. , and Huang Y. , et al.Probiotics and Paraprobiotics in Aquaculture: A Sustainable Strategy for Enhancing Fish Growth, Health and Disease Prevention-a Review, Frontiers in Marine Science. (2024) 11, 10.3389/fmars.2024.1499228, 1499228.

[103] Kalita R. , Pegu A. , and Baruah C. , Prospects of Probiotics and Fish Growth Promoting Bacteria in Aquaculture: A Review, International Journal of Agriculture and Biosciences. (2023) 12, no. 4, 234–244, 10.47278/journal.ijab/2023.070.

[104] Huang X. , He H. , and Li Z. , et al.Screening and Effects of Intestinal Probiotics on Growth Performance, Gut Health, Immunity, and Disease Resistance of Nile Tilapia (*Oreochromis niloticus*) Against *Streptococcus agalactiae* , Fish & Shellfish Immunology. (2024) 151, 10.1016/j.fsi.2024.109668, 109668. 38838837

[105] Hai N. V. , The Use of Probiotics in Aquaculture, Journal of Applied Microbiology. (2015) 119, no. 4, 917–935, 10.1111/jam.12886. 26119489

[106] López Nadal A. , Ikeda-Ohtsubo W. , and Sipkema D. , et al.Feed, Microbiota, and Gut Immunity: Using the Zebrafish Model to Understand Fish Health, Frontiers in Immunology. (2020) 11, 10.3389/fimmu.2020.00114, 114. 32117265 PMC7014991

[107] Karimi R. , Homayoonfal M. , Malekjani N. , Kharazmi M. S. , and Jafari S. M. , Interaction Between β-Glucans and Gut Microbiota: A Comprehensive Review, Critical Reviews in Food Science and Nutrition. (2024) 64, no. 22, 7804–7835, 10.1080/10408398.2023.2192281. 36975759

[108] Singla A. , Gupta O. P. , and Sagwal V. , et al.Beta-Glucan as a Soluble Dietary Fiber Source: Origins, Biosynthesis, Extraction, Purification, Structural Characteristics, Bioavailability, Biofunctional Attributes, Industrial Utilization, and Global Trade, Nutrients. (2024) 16, no. 6, 10.3390/nu16060900, 900. 38542811 PMC10975496

[109] Yang P. J. , Zhan M. Y. , Ye C. , Yu X. Q. , and Rao X. J. , Molecular Cloning and Characterization of a Short Peptidoglycan Recognition Protein From Silkworm *Bombyx mori* , Insect Molecular Biology. (2017) 26, no. 6, 665–676, 10.1111/imb.12330. 28703893

[110] Yang Y. , Lim J. , Li C. , Lee S. , and Hong S. , Effects of Sulfated Polysaccharides Isolated From *Codium fragile* on Inflammatory Cytokine Gene Expression and *Edwardsiella tarda* Infection in Rockfish, *Sebastes schlegelii* , Fish & Shellfish Immunology. (2021) 112, 125–134, 10.1016/j.fsi.2021.03.001. 33737238

[111] Jiang C. , Xu M. , and Kuang X. , et al. *Treponema pallidum* Flagellins Stimulate MMP-9 and MMP-13 Expression via TLR5 and MAPK/NF-κB Signaling Pathways in Human Epidermal Keratinocytes, Experimental Cell Research. (2017) 361, no. 1, 46–55, 10.1016/j.yexcr.2017.09.040. 28982539

[112] Gao Q. , Gao Q. , Min M. , Zhang C. , Peng S. , and Shi Z. , Ability of Lactobacillus Plantarum Lipoteichoic Acid to Inhibit Vibrio Anguillarum-Induced Inflammation and Apoptosis in Silvery Pomfret (Pampus Argenteus) Intestinal Epithelial Cells, Fish & Shellfish Immunology. (2016) 54, 573–579. 27179425 10.1016/j.fsi.2016.05.013

[113] Gao Q. , Yue Y. , and Min M. , et al.Characterization of TLR5 and TLR9 From Silver Pomfret (*Pampus argenteus*) and Expression Profiling in Response to Bacterial Components, Fish & Shellfish Immunology. (2018) 80, 241–249, 10.1016/j.fsi.2018.06.014. 29890218

[114] Abdel-Kader M. F. , Shukry M. , and Dawood M. A. , et al.Ameliorative Effect of Dietary Lipopolysaccharides on *Oreochromis niloticus* Juveniles Submitted to Aflatoxin B1-Induced Oxidative Stress and Bacterial Challenge, Aquaculture Research. (2021) 52, no. 8, 3660–3676, 10.1111/are.15211.

[115] Holen E. , Austgulen M. H. , and Espe M. , RNA Form Baker’s Yeast Cultured With and Without Lipopolysaccharide (LPS) Modulates Gene Transcription in an Intestinal Epithelial Cell Model, RTgutGC From Rainbow Trout (*Oncorhynchus mykiss*), Fish & Shellfish Immunology. (2021) 119, 397–408, 10.1016/j.fsi.2021.10.018. 34687880

[116] Ali M. F. Z. , Nakahara S. , Otsu Y. , Ido A. , Miura C. , and Miura T. , Effects of Functional Polysaccharide From Silkworm as an Immunostimulant on Transcriptional Profiling and Disease Resistance in Fish, Journal of Insects as Food and Feed. (2022) 8, no. 11, 1221–1233, 10.3920/JIFF2021.0108.

[117] Nya E. J. and Austin B. , Use of Bacterial Lipopolysaccharide (LPS) as an Immunostimulant for the Control of *Aeromonas hydrophila* Infections in Rainbow Trout *Oncorhynchus mykiss* (Walbaum), Journal of Applied Microbiology. (2010) 108, no. 2, 686–694, 10.1111/j.1365-2672.2009.04464.x. 19674184

[118] Mo W. Y. , Cheng Z. , and Choi W. M. , et al.Use of Food Waste as Fish Feeds: Effects of Prebiotic Fibers (Inulin and Mannanoligosaccharide) on Growth and Non-Specific Immunity of Grass Carp (*Ctenopharyngodon idella*), Environmental Science and Pollution Research. (2015) 22, no. 22, 17663–17671, 10.1007/s11356-015-4971-z. 26150295

[119] Mohan K. , Muralisankar T. , and Uthayakumar V. , et al.Trends in the Extraction, Purification, Characterisation and Biological Activities of Polysaccharides From Tropical and Sub-Tropical Fruits–A Comprehensive Review, Carbohydrate Polymers. (2020) 238, 10.1016/j.carbpol.2020.116185, 116185. 32299552

[120] Motta F. , Gershwin M. E. , and Selmi C. , Mushrooms and Immunity, Journal of Autoimmunity. (2021) 117, 10.1016/j.jaut.2020.102576, 102576. 33276307

[121] Moroni F. , Naya-Català F. , and Piazzon M. C. , et al.The Effects of Nisin-Producing *Lactococcus lactis* Strain Used as Probiotic on Gilthead Sea Bream (*Sparus aurata*) Growth, Gut Microbiota, and Transcriptional Response, Frontiers in Marine Science. (2021) 8, 10.3389/fmars.2021.659519, 659519.

[122] Dawood M. A. O. , Koshio S. , and Ishikawa M. , et al.Dietary Supplementation of β-Glucan Improves Growth Performance, the Innate Immune Response and Stress Resistance of Red Sea Bream, *Pagrus major* , Aquaculture Nutrition. (2017) 23, no. 1, 148–159, 10.1111/anu.12376.

[123] Abu-Elala N. M. , El-Sayed Ali T. , and Ragaa N. M. , et al.Analysis of the Productivity, Immunity, and Health Performance of Nile Tilapia (*Oreochromis niloticus*) Broodstock-Fed Dietary Fermented Extracts Sourced From *Saccharomyces cerevisiae* (Hilyses): A Field Trial, Animals. (2021) 11, no. 3, 10.3390/ani11030815, 815. 33799378 PMC7998373

[124] Mahdy M. A. , Jamal M. T. , Al-Harb M. , Al-Mur B. A. , and Haque M. F. , Use of Yeasts in Aquaculture Nutrition and Immunostimulation: A Review, Journal of Applied Biology & Biotechnology. (2022) 10, 59–65, 10.7324/JABB.2022.100507.

[125] Zhou X. , Notes From the Aquaculture Statistician, 2018, 6–8, FAO Aquaculture Newsletter 58.

[126] Øverland M. and Skrede A. , Yeast Derived from Lignocellulosic Biomass as a Sustainable Feed Resource for use in Aquaculture, Journal of the Science of Food and Agriculture. (2017) 97, no. 3, 733–742, 10.1002/jsfa.8007. 27558451

[127] Meena D. K. , Das P. , and Kumar S. , et al.Beta-Glucan: An Ideal Immunostimulant in Aquaculture (A Review), Fish Physiology and Biochemistry. (2013) 39, no. 3, 431–457, 10.1007/s10695-012-9710-5. 22965949

[128] Vargas-Albores F. , Martínez-Porchas M. , and Gollas-Galván T. , Yeast-Derived Immunostimulants in Fish and Shrimp Aquaculture: Molecular Insights and Applications, Frontiers in Immunology. (2023) 14, 1221348.

[129] Selvaraj V. , Sampath K. , and Sekar V. , Adjuvant and Immunostimulatory Effects of β-Glucan Administration in Combination With Lipopolysaccharide Enhances Survival and Some Immune Parameters in Carp Challenged With Aeromonas hydrophila, Veterinary Immunology and Immunopathology. (2006) 114, no. 1-2, 15–24. 16919782 10.1016/j.vetimm.2006.06.011

[130] Hamza F. and Zinjarde S. , Use of Marine Microorganisms in Designing Anti-Infective Strategies for Sustainable Aquaculture Production, Journal of Applied Microbiology. (2023) 134, no. 7. 10.1093/jambio/lxad128 37365690

[131] Hadiuzzaman M. , Moniruzzaman M. , Shahjahan M. , Bai S. C. , Min T. , and Hossain Z. , β-Glucan: Mode of Action and Its Uses in Fish Immunomodulation, Frontiers in Marine Sciences. (2022) 9, 10.3389/fmars.2022.905986, 905986.

[132] Torrecillas S. , Montero D. , and Izquierdo M. , Improved Health and Growth of Fish Fed Mannan Oligosaccharides: Potential Mode of Action, Fish & Shellfish Immunology. (2014) 36, no. 2, 525–544, 10.1016/j.fsi.2013.12.029. 24412165

[133] White W. L. and Wilson P. , World Seaweed Utilization, Seaweed Sustainability, 2015, Academic Press, 7–25.

[134] Leandro A. , Pereira L. , and Gonçalves A. M. , Diverse Applications of Marine Macroalgae, Marine Drugs. (2020) 18, no. 1, 10.3390/md18010017, 17. PMC7024196 31878264

[135] Vijayaram S. , Ringø E. , Ghafarifarsani H. , Hoseinifar S. H. , Ahani S. , and Chou C. C. , Use of Algae in Aquaculture: A Review, Fishes. (2024) 9, no. 2, 63.

[136] Al-Wakeel A. H. , Elbahnaswy S. , Risha E. , and Zahran E. , Dietary *Pediastrum boryanum* Microalgal Extract Improves Growth, Enhances Immunity, and Regulates Immune-Related Genes in Nile Tilapia, BMC Veterinary Research. (2024) 20, no. 1, 10.1186/s12917-024-04155-z, 321. 39026262 PMC11256681

[137] Arteaga Quico C. , Mariano Astocondor M. , and Aquino Ortega R. , Dietary Supplementation With *Chlorella peruviana* Improve the Growth and Innate Immune Response of Rainbow Trout *Oncorhynchus mykiss* Fingerlings, Aquaculture. (2021) 533, 10.1016/j.aquaculture.2020.736117, 736117.

[138] Sánchez F. , Lozano-Muñoz I. , Muñoz S. , Diaz N. , Neira R. , and Wacyk J. , Effect of Dietary Inclusion of Microalgae (*Nannochloropsis gaditana* and *Schizochytrium spp*) on Non-Specific Immunity and Erythrocyte Maturity in Atlantic Salmon Fingerlings, Fish & Shellfish Immunology. (2023) 140, 10.1016/j.fsi.2023.108975, 108975. 37488040

[139] Ferreira M. , Machado M. , and Mota C. S. C. , et al.Micro- and Macroalgae Blend Modulates the Mucosal and Systemic Immune Responses of European Seabass (*Dicentrarchus labrax*) Upon Infection With *Tenacibaculum maritimum* , Aquaculture. (2023) 566, 10.1016/j.aquaculture.2022.739222, 739222.

[140] Abdelmohsen T. A. , Saad A. H. , Al wakeel R. A. , Fadl S. E. , and Hamouda A. H. , Effect of Plant and Microalgae on Immune-Related Genes and Resistance of Nile Tilapia (*Oreochromis niloticus*) against *Aeromonas hydrophila* , Scientific Reports. (2025) 15, no. 1, 10.1038/s41598-025-09715-3, 27564. 40730597 PMC12307586

[141] Méndez-Vivancos F. , Sáez M. I. , and Galafat A. , et al.Assessment of the Immunomodulatory Effect of Using Raw or Hydrolysed *Nannochloropsis gaditana* in Diets for Juvenile Gilthead Seabream Specimens, Aquaculture Reports. (2025) 45, 10.1016/j.aqrep.2025.103163, 103163.

[142] Marmelo I. , Dias M. , and Grade A. , et al.Immunomodulatory and Antioxidant Effects of Functional Aquafeeds Biofortified With Whole *Laminaria digitata* in Juvenile Gilthead Seabream (*Sparus aurata*), Frontiers in Marine Science. (2024) 11, 10.3389/fmars.2024.1325244, 1325244.

[143] García-Beltrán J. M. , Arizcun M. , and Chaves-Pozo E. , Antimicrobial Peptides From Photosynthetic Marine Organisms With Potential Application in Aquaculture, Marine Drugs. (2023) 21, no. 5, 10.3390/md21050290, 290. 37233484 PMC10222056

[144] Ringø E. , Ashour M. , Ahmed S. , Sharawy Z. , Goda A. , and El-Haroun E. , Severo I. A. , Microalgae and Seaweeds as Feed Additives for Aquatic Animals: Effects on Growth, Immunity, and Disease Resistance, Algae – Science and Applications, 2025, IntechOpen, 79–101.

[145] Mahmoud M. M. A. , El-Lamie M. M. M. , Kilany O. E. , and Dessouki A. A. , Spirulina (*Arthrospira platensis*) Supplementation Improves Growth Performance, Feed Utilization, Immune Response, and Relieves Oxidative Stress in Nile Tilapia (*Oreochromis niloticus*) Challenged With *Pseudomonas fluorescens* , Fish & Shellfish Immunology. (2018) 72, 291–300, 10.1016/j.fsi.2017.11.006. 29117593

[146] Sheikhzadeh N. , Mousavi S. , Hamidian G. , Firouzamandi M. , Oushani A. K. , and Mardani K. , Role of Dietary *Spirulina platensis* in Improving Mucosal Immune Responses and Disease Resistance of Rainbow Trout (*Oncorhynchus mykiss*), Aquaculture. (2019) 510, 1–8, 10.1016/j.aquaculture.2019.05.009.

[147] Rahman M. , Mamun M. A. , and Rathore S. S. , et al.Effects of Supplementation of Natural *Spirulina* on Growth Performance, Hemato-Biochemical Indices, Gut Health and Disease Resistance to *Aeromonas hydrophila* of Stinging Catfish (*Heteropneustes fossilis*) Fingerlings, Aquaculture Reports. (2023) 32, 10.1016/j.aqrep.2023.101727, 101727.

[148] Simjee S. and Ippolito G. , European Regulations on Prevention use of Antimicrobials From January 2022, Brazilian Journal of Veterinary Medicine. (2022) 44, 10.29374/2527-2179.bjvm000822, e000822. 36225552 PMC9543772

[149] Reverter M. , Tapissier-Bontemps N. , Sarter S. , Sasal P. , and Caruso D. , Moving Towards More Sustainable Aquaculture Practices: A Meta-Analysis on the Potential of Plant-Enriched Diets to Improve Fish Growth, Immunity and Disease Resistance. Reviews in Aquaculture. (2021) 13, no. 1, 537–555, 10.1111/raq.12485.

[150] Egerton S. , Culloty S. , Whooley J. , Stanton C. , and Ross R. P. , The Gut Microbiota of Marine Fish, Frontiers in Microbiology. (2018) 9, 10.3389/fmicb.2018.00873, 873. 29780377 PMC5946678

[151] Liao Z. and Su J. , Progresses on Three Pattern Recognition Receptor Families (TLRs, RLRs and NLRs) in Teleost, Developmental & Comparative Immunology. (2021) 122, 10.1016/j.dci.2021.104131, 104131. 34022258

[152] Ponce M. , Zuasti E. , Anguís V. , and Fernández-Díaz C. , Anti-Bacterial and Immunostimulatory Properties of Ulvan-Loaded Chitosan Nanoparticles for Use in Aquaculture, Marine Biotechnology. (2024) 26, no. 1, 19–27, 10.1007/s10126-023-10272-x. 38110743

